# Potential benefit of extended dose schedules of human papillomavirus vaccination in the context of scarce resources and COVID-19 disruptions in low-income and middle-income countries: a mathematical modelling analysis

**DOI:** 10.1016/S2214-109X(22)00475-2

**Published:** 2022-12-13

**Authors:** Élodie Bénard, Mélanie Drolet, Jean-François Laprise, Mark Jit, Kiesha Prem, Marie-Claude Boily, Marc Brisson

**Affiliations:** a Département de médecine sociale et préventive, Université Laval, QC, Canada; b Centre de recherche du CHU de Québec, Université Laval, QC, Canada; c Centre for Mathematical Modelling of Infectious Disease, London School of Hygiene and Tropical Medicine, London, UK; d School of Public Health, University of Hong Kong, Hong Kong Special Administrative Region, China; e Saw Swee Hock School of Public Health, National University of Singapore, Singapore; f MRC Centre for Global Infectious Disease Analysis, School of Public Health, Imperial College London, London, UK

## Abstract

**Background:**

The WHO Strategic Advisory Group of Experts recommended that an extended interval of 3–5 years between the two doses of the human papillomavirus (HPV) vaccine could be considered to alleviate vaccine supply shortages. However, three concerns have limited the introduction of extended schedules: girls could be infected between the two doses, the vaccination coverage for the second dose could be lower at ages 13–14 years than at ages 9–10 years, and identifying girls vaccinated with a first dose to give them the second dose could be difficult. Using mathematical modelling, we examined the potential effect of these concerns on the population-level impact and efficiency of extended dose HPV vaccination schedules.

**Methods:**

We used HPV-ADVISE, an individual-based, transmission-dynamic model of multitype HPV infection and disease, calibrated to country-specific data for four low-income and middle-income countries (India, Viet Nam, Uganda, and Nigeria). For the extended dose scenarios, we varied the vaccination coverage of the second dose among girls previously vaccinated, the one-dose vaccine efficacy, and the one-dose vaccine duration of protection. We also examined a strategy in which girls aged 14 years were vaccinated irrespective of their previous vaccination status. We used a scenario of girls-only two-dose vaccination at age 9 years (vaccine=9 valent, vaccine-type efficacy=100%, duration of protection=lifetime, and coverage=80%) as our comparator. We estimated two outcomes: the relative reduction in the age-standardised cervical cancer incidence (population-level impact) and the number of cervical cancers averted per 100 000 doses (efficiency).

**Findings:**

Our model projected substantial reductions in cervical cancer incidence over 100 years with the two-dose schedule (79–86% depending on the country), compared with no vaccination. Projections for the 5-year extended schedule, in which the second dose is given only to girls previously vaccinated at age 9 years, were similar to the current two-dose schedule, unless vaccination coverage of the second dose is very low (reductions in cervical cancer incidence of 71–78% assuming 30% coverage at age 14 years among girls vaccinated at age 9 years). However, when the dose at age 14 years is given to girls irrespective of vaccination status and assuming high vaccination coverage, the model projected a substantially greater reduction in cervical cancer incidence compared with the current two-dose schedule (reductions in cervical cancer incidence of 86–93% assuming 70% coverage at age 14 years, irrespective of vaccination status). Efficiency of the extended schedule was greater than the two-dose schedule, even with a drop in vaccination coverage.

**Interpretation:**

The three concerns are unlikely to have a substantial effect on the population-level impact of extended dose schedules. Hence, extended dose schedules will likely provide similar cervical cancer reductions as two-dose schedules, while reducing the number of doses required in the short-term, providing a more efficient use of scarce resources, and offering a 5-year time window to reassess the necessity of the second dose.

**Funding:**

WHO, Canadian Institute of Health Research Foundation, Fonds de recherche du Québec–Santé, Digital Research Alliance of Canada, and Bill & Melinda Gates Foundation.

## Introduction

In 2019, the WHO Strategic Advisory Group of Experts recommended that an extended interval of 3–5 years between the two doses of the human papillomavirus (HPV) vaccine (first dose given around ages 9–10 years and the second dose around ages 13–14 years) could be considered when introducing HPV vaccination in a country to alleviate HPV vaccine supply shortage.^1^ The recommendation was based on studies indicating that geometrical mean IgG antibody titres were similar when the second dose of the HPV vaccine was given 6 months or 3–8 years after the first dose,^2^ and on a modelling study showing that an extended schedule could provide similar population-level impact against cervical cancer as the current two-dose schedule, while minimising short-term vaccine demand and costs.^3^ Based on these results, the UK and Québec, Canada, have implemented extended HPV vaccination schedules.^4^, ^5^ Furthermore, the recommendation of an extended schedule is particularly relevant during the COVID-19 pandemic. Globally, HPV vaccination programmes have been severely disrupted due to physical distancing measures (eg, lockdowns and school closures), health system constraints (eg, reassignment of health-care workers), and worries of risk of transmission during immunisation visits.^6^

**Research in context**

**Evidence before this study**

We have previously shown, using mathematical modelling, that a 5-year extended schedule is likely to be highly efficient and cost-effective in low-income and middle-income countries. In 2019, partly based on these results, the WHO Strategic Advisory Group of Experts recommended that an extended interval of 3–5 years between the two doses of the human papillomavirus (HPV) vaccine (first dose around ages 9–10 years, second dose around ages 13–14 years) could be considered when introducing HPV vaccination in a country. This recommendation is particularly relevant during the COVID-19 pandemic, because organising HPV routine vaccination with two doses in the same year might represent a major challenge given resource constraints, which have been exacerbated by the COVID-19 pandemic. Moreover, there is increasing evidence of the high efficacy and durability of a single dose of HPV vaccines. However, three concerns have limited the widespread introduction of HPV vaccination extended schedules: (1) girls could be infected between the two doses, if they become sexually active and one-dose vaccine efficacy is limited; (2) vaccination coverage for the second dose could be much lower if scheduled at ages 13 or 14 years in countries where school attendance substantially drops with age; (3) and implementation challenges of finding the girls to administer the second dose several years after the first dose, although there might be alternative strategies, such as catch-up campaigns irrespective of vaccine status. We searched PubMed (no date restriction) and found no modelling study that have examined the potential impact of extended schedules considering these concerns.

**Added value of this study**

In this modelling analysis, we showed that: girls becoming sexually active between the two doses should be, at the very least, partially protected by one dose; (2) reductions in coverage for the second dose in an extended dose schedule at ages 13–14 years is unlikely to have a substantial effect on the number of cases of cervical cancer prevented, unless vaccination coverage of the second dose is very low (eg, 30%) or one-dose vaccine efficacy is of short duration; and (3) alternative strategies to finding previously vaccinated girls, such as catch-up campaigns in which girls are vaccinated at age 14 years irrespective of their vaccination status, could provide an opportunity to vaccinate girls who were missed at age 9 years, thus increasing population-level impact. Hence, extended dose schedules would likely provide similar cervical cancer reductions as two-dose schedules, while reducing the number of doses required in the short-term and providing a more efficient use of scarce vaccine resources.

**Implications of all the available evidence**

These findings have important policy implications because they show that the concerns about extended schedules should not prevent countries from introducing such strategies, particularly in the context of the COVID-19 pandemic. An extended dose schedule could offer an effective and efficient strategy of HPV vaccination while providing time to reassess whether it is necessary to give the second dose. In fact, with the increasing evidence showing high efficacy and durability of a single dose, the province of Québec (Canada) and England (UK) have adopted such a strategy. Moreover, an extended schedule with a catch-up campaign at ages 13 or 14 years, in which girls are vaccinated with one dose, irrespective of whether they were previously vaccinated at age 9 years, could provide the opportunity to vaccinate a greater number of girls with at least one dose and therefore increase the population-level impact of HPV vaccination. Finally, an extended schedule might be easier to implement with the WHO's 2022 recommendation that one-dose or two-dose schedules could be considered for girls aged 9–14 years, as it could provide a security net for countries who are uncertain about starting directly with or switching to a one-dose strategy.

Although we have previously shown that a 5-year extended HPV vaccination schedule was likely to be highly efficient and cost effective in low-income and middle-income countries (LMICs),^7^ three main concerns have limited the widespread introduction of HPV vaccination extended schedules. First, there is a concern that girls could be more likely to be infected between the first and second dose if they become sexually active and if the one-dose vaccine efficacy is limited. Second, there is a worry that vaccination coverage for the second dose could be much lower if scheduled to be given at ages 13 or 14 years in countries where school attendance substantially drops with age. Third, there are implementation challenges of finding the girls to administer the second dose several years after the first dose, although there might be alternative strategies, such as catch-up campaigns irrespective of vaccine status (eg, one dose at age 9 years and one dose at age 14 years).

The objective of this modelling study was to examine the concerns about the extended schedules of HPV vaccinations and their potential effect on population-level impact (reduction of cervical cancer incidence) and vaccination efficiency (number of cervical cancer cases prevented per 100 000 vaccine doses). To do so, we used a transmission-dynamic model to compare the population-level impact and efficiency of 5-year extended HPV vaccination schedules to the current two-dose recommended schedule at age 9 years, for different assumptions of vaccination coverage and one-dose vaccine efficacy and duration of protection, using India, Viet Nam, Uganda, and Nigeria as examples.

## Methods

### Model description

We used HPV-ADVISE LMIC, an individual-based, transmission-dynamic model of multitype HPV infection and diseases (appendix 1).^7^ The model has five fully integrated components: sociodemographic characteristics, sexual behaviour and HPV transmission, HPV-related diseases, vaccination, and screening and treatment. 18 HPV types, including all types in the 9-valent vaccine, are modelled individually and independently. Each HPV type has its own natural history parameters in terms of transmission, persistence, clearance, and disease progression to cervical cancer. The model simulates type-specific HPV transmission through sexual activity (based on different risk groups and sexual mixing) and type-specific natural history of cervical cancer, from persistent HPV infection to precancerous lesions and cervical cancer. The model assumes that HPV vaccines are prophylactic and do not alter the natural history of HPV among individuals infected at the time of vaccination.^8^ HPV-ADVISE LMIC is implemented in C++ (version 11).

We modelled the impact of the HPV vaccination in two Asian countries (India and Viet Nam) and two African countries (Uganda and Nigeria) to represent different profiles of sexual activity and HPV-related burden (appendix 2 p 3).^7^ We parameterised and calibrated the model to each of the four countries separately. The parameter values for sexual behaviour and natural history of HPV and cervical cancer were identified through calibration to highly stratified sexual behaviour (ie, age-specific rates of sexual debut and lifetime number of partners) and epidemiological data (ie, age-specific HPV prevalence and cervical cancer incidence) from India, Viet Nam, Uganda, and Nigeria (appendix 1 pp 6–46). For each country, we identified 50 parameter sets that simultaneously fit country-specific behavioural and epidemiological data (appendix 1 pp 47–51). These 50 parameter sets show the uncertainty and variability in sexual behaviour and HPV epidemiology within each country. Reporting was done according to HPV-FRAME, a consensus-based framework for modelled evaluations of HPV prevention and cervical cancer control (appendix 2 pp 4–5).^9^

### Vaccination scenarios

**Figure 1:**
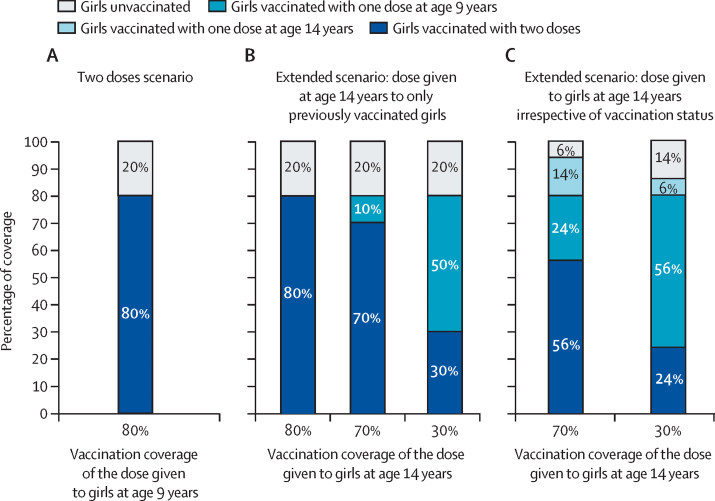
Assumed vaccination coverage for three scenarios of vaccination of girls at ages 9 and 14 years (A) Current two-dose vaccination schedule at age 9 years. (B) 5-year extended two-dose schedule (first dose at age 9 years and second dose at age 14 years) where the second dose is given to previously vaccinated girls only. (C) 5-year extended two-dose schedule (first dose at age 9 years and second dose at age 14 years) where the second dose is given at age 14 years irrespective of vaccination status.

**Figure 2:**
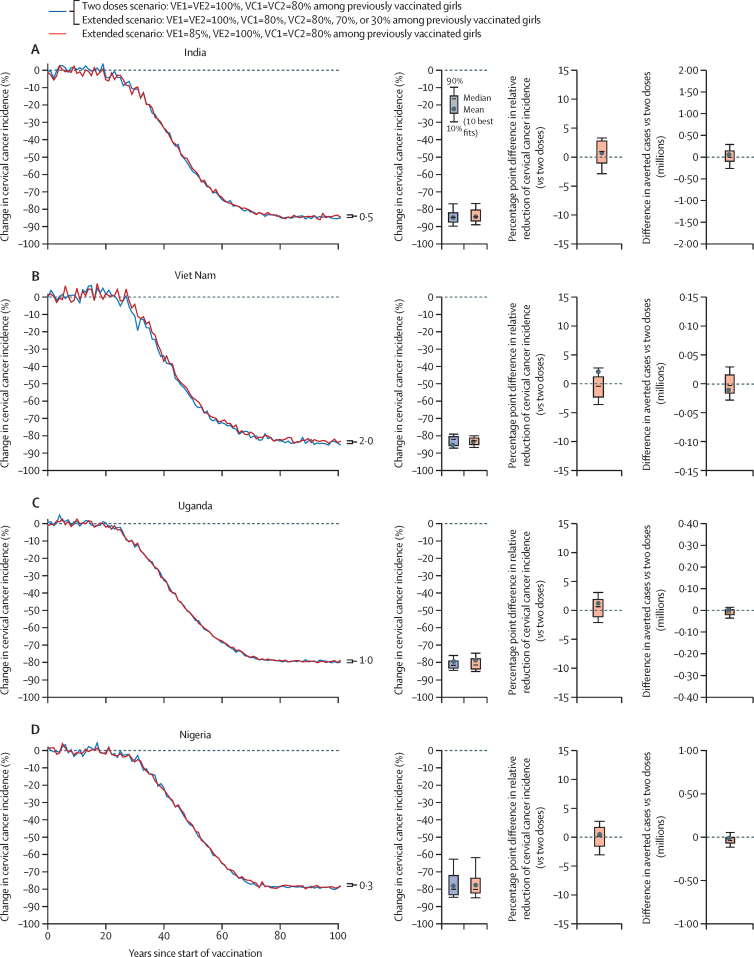
Projected population-level impact of the current two-dose schedule and a 5-year extended two-dose schedule with lower efficacy of one dose in India (A), Viet Nam (B), Uganda (C), and Nigeria (D) In this extended scenario, the second dose is given to previously vaccinated girls only. When assuming a 100% vaccine efficacy of the first dose, the results are identical for the different HPV vaccination second-dose coverage scenarios (30%, 70%, or 80%). The lines represent the mean of the ten best fitting parameter sets to the incidence of cervical cancer from the Global Cancer Observatory 2020. Uncertainty intervals should not be interpreted as confidence interval from a statistical point of view. Uncertainty intervals reflect uncertainty in model parameters and variability in HPV epidemiology within a country. To compare the results between vaccination strategies, the uncertainty intervals around the following outcomes should be used: percentage point difference in relative reduction of cervical cancer (*vs* two doses) and difference in averted cases (*vs* two doses). VE1=vaccine efficacy of dose 1. §VE2=vaccine efficacy of dose 2. VC1=vaccination coverage of dose 1. VC2=vaccination coverage of dose 2. Vaccine duration of protection after one dose=lifelong for all scenarios. HPV=human papillomavirus.

We reproduced three different girls-only vaccination scenarios to examine the main concerns of extended schedules using the 9-valent HPV vaccine: (1) a current two-dose vaccination schedule given at age 9 years (figure 1A); (2) a 5-year extended two-dose schedule (first dose given at age 9 years and a second dose at age 14 years) where the second dose is given to previously vaccinated girls only (figure 1B); and (3) a 5-year extended two-dose schedule (first dose given at age 9 years and a second dose at age 14 years) where the second dose is given to girls at age 14 years irrespective of previous vaccination status (figure 1C). The third scenario would provide the opportunity to vaccinate, with one dose, girls at age 14 years who might have been missed being vaccinated at age 9 years.

For all scenarios, vaccination coverage at age 9 years was assumed to be 80% on the basis of estimates of the median vaccination coverage for the first dose observed in LMICs in 2019.^10^ There are examples of LMICs that have introduced HPV vaccination of girls aged 14 years (as routine vaccination or as part of a catch-up) and were able to reach high vaccination coverage (eg, in Tanzania, Rwanda, and Ethiopia).^10^, ^11^, ^12^ However, given the uncertainty about the coverage that can be reached for girls aged 14 years because of the potential drop in school attendance, we examined three levels of vaccination coverage: 30%, 70%, and 80%. We chose 30% as our pessimistic scenario of vaccination coverage because it is among the lowest coverages observed for the second dose in LMICs with HPV vaccination programmes that reached 70–90% for the first dose in 2019.^10^ In the base-case analysis, we assumed that two doses provide 100% efficacy and lifetime duration of protection, and one dose provides 85% or 100% efficacy, based on the range of estimates from the KEN SHE Study and the India IARC Trial.^13^, ^14^

### Sensitivity analysis

In our sensitivity analysis, we chose 20 years of duration of protection following one-dose vaccination as a pessimistic scenario, as results from the India IARC Trial show sustained protection of one dose through 10 years.^13^ We would have already observed a decline in protection if the average one-dose duration of protection was 20 years or less. To show the potential effect of lower coverage at age 9 years, we also modelled a 65% vaccination coverage at age 9 years for the current two-dose and the extended schedules (where the second dose is given irrespective of previous vaccination status). For this extended schedule, we modelled two scenarios of vaccination coverage at age 14 years (65% and 50%). These vaccination coverages were based on the average estimates for the first (67%) and second (53%) doses among all countries with HPV vaccination programmes in 2019.^10^

### Outcomes

We used two main outcomes. To examine the population-level impact of the different HPV vaccination scenarios, we used the relative reduction in cervical cancer incidence compared with no vaccination. To examine the efficiency of the vaccination scenarios, we calculated the number of cervical cancers averted over time per 100 000 doses given. For both outcomes, we present the mean of the ten best fitting parameter sets for cervical cancer incidence from 2020 from the Global Cancer Observatory^15^ to provide an estimate that represents recent average national cervical cancer incidence estimates. Model projections are also presented with the 10th and 90th percentiles (80% uncertainty interval) obtained from 1000 simulations for each scenario (50 parameter sets × 20 simulations) to represent uncertainty and variability in HPV epidemiology and sexual behaviour within a country (eg, for Viet Nam^16^ and India,^17^ cervical cancer incidence varies substantially within the country). To capture the short-term and long-term impact of vaccination, the time horizon was set to 100 years. We calculated cervical cancer averted using age-specific and country-specific population projections from 2020 to 2100 from the UN World Population Prospects, and we extrapolated these demographic projections from 2100 to 2120 (appendix 2 p 20).^18^

### Role of the funding source

The funder of the study had no role in study design, data collection, data analysis, data interpretation, or writing of the report.

## Results

Under base-case assumptions for the current two-dose strategy (80% vaccination coverage, two-dose efficacy=100%, duration=lifelong), the model projected that vaccinating girls at age 9 years would produce substantial reductions in cervical cancer incidence by 85% in India, 86% in Viet Nam, 80% in Uganda, and 79% in Nigeria, after 100 years (figure 2, appendix 2 pp 6–13). As expected, if one dose provides the same short-term and long-term efficacy as two doses, then a 5-year extended schedule with the second dose given to previously vaccinated girls only would produce the same population-level impact as the current two-dose strategy irrespective of the second dose coverage (ie, the second dose would be redundant; figure 2).

When assuming lower vaccine efficacy for one dose (one-dose efficacy=85%, two-dose efficacy=100%) and a high vaccination coverage for both doses (80% vaccination coverage), the model projected that a 5-year extended schedule would produce similar reductions in cervical cancer incidence in the four countries compared with the current two-dose strategy (0·3 to 2·0 percentage point difference in the relative reduction of cervical cancer incidence at equilibrium; figure 2). Therefore, the start of sexual activity and potential infection between the first and second dose in an extended schedule would have little effect on the overall population-level impact of HPV vaccination.

**Figure 3:**
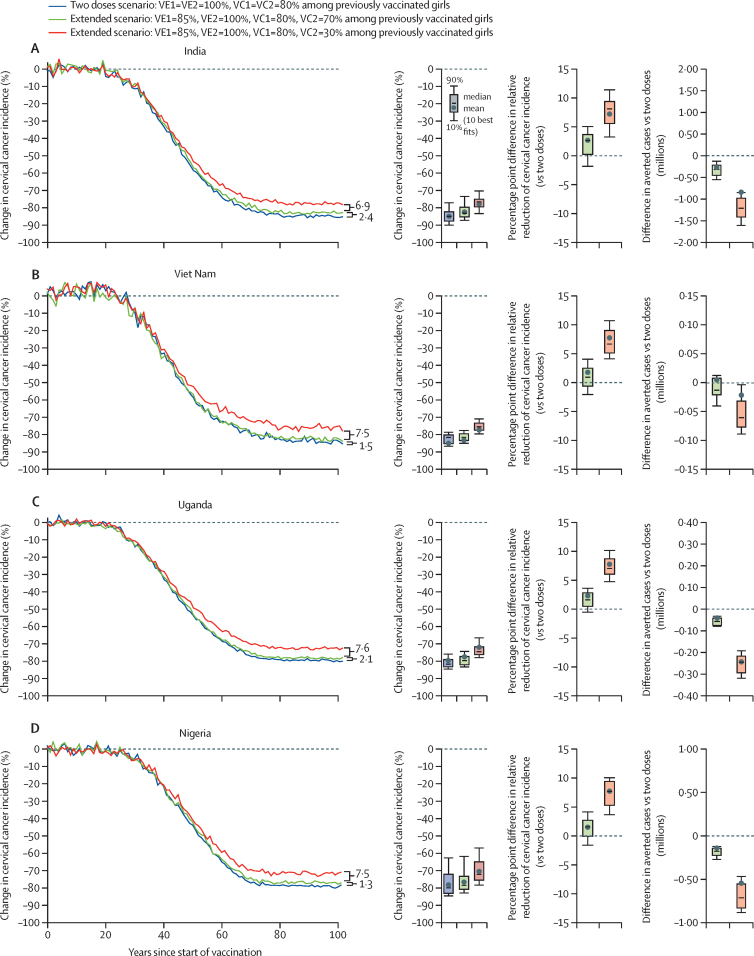
Projected population-level impact of a 5-year extended two-dose schedule, with lower coverage for the second dose among previously vaccinated girls in India (A), Viet Nam (B), Uganda (C), and Nigeria (D) In this extended scenario, the second dose is given to previously vaccinated girls only. The lines represent the mean of the ten best fitting parameter sets to the incidence of cervical cancer from the Global Cancer Observatory 2020. Uncertainty intervals should not be interpreted as confidence interval from a statistical point of view. Uncertainty intervals reflect uncertainty in model parameters and variability in HPV epidemiology within a country. To compare the results between vaccination strategies, the uncertainty intervals around the following outcomes should be used: percentage point difference in relative reduction of cervical cancer (*vs* two doses) and difference in averted cases (*vs* two doses). VE1=vaccine efficacy of dose 1. VE2=vaccine efficacy of dose 2. VC1=vaccination coverage of dose 1. VC2=vaccination coverage of dose 2. Vaccine duration of protection after one dose=lifelong for all scenarios. HPV=human papillomavirus.

When assuming lower vaccine efficacy for one dose (one-dose efficacy=85%, two-dose efficacy=100%) and a 70% vaccination coverage for the second dose at age 14 years (80% for the first dose), the model projected that a 5-year extended schedule with the second dose given to previously vaccinated girls only would result in slightly more cervical cancer cases in the four countries than the current two-dose strategy with 80% vaccination coverage (1·3 to 2·4 percentage point difference in the relative reduction of cervical cancer incidence at equilibrium; figure 3; appendix 2 pp 6–13). However, if the second dose vaccination coverage drops to 30%, an extended schedule would result in substantially more cervical cancer cases in the four countries than the current two-dose schedule (6·9 to 7·6 percentage point difference in the relative reduction of cervical cancer incidence at equilibrium; figure 3).

**Figure 4:**
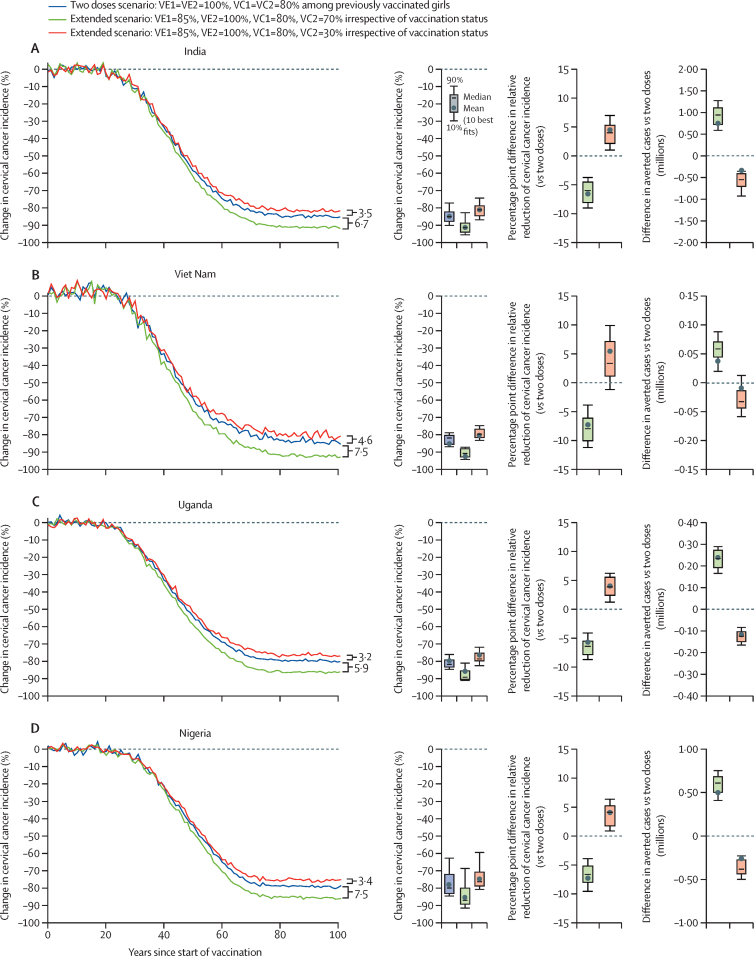
Projected population-level impact of a 5-year extended two-dose schedule, where the second dose is given at age 14 years, irrespective of vaccination status, in India (A), Viet Nam (B), Uganda (C), and Nigeria (D) In this extended scenario, the dose is given to girls age at 14 years irrespective of vaccination status. The lines represent the mean of the 10 best fitting parameter sets to the incidence of cervical cancer from the Global Cancer Observatory 2020. Uncertainty intervals should not be interpreted as confidence interval from a statistical point of view. Uncertainty intervals reflect uncertainty in model parameters and variability in HPV epidemiology within a country. To compare the results between vaccination strategies, the uncertainty intervals around the following outcomes should be used: percentage point difference in relative reduction of cervical cancer (*vs* two doses) and difference in averted cases (*vs* two doses). VE1=vaccine efficacy of dose 1. VE2=vaccine efficacy of dose 2. VC1=vaccination coverage of dose 1. VC2=vaccination coverage of dose 2. Vaccine duration of protection after one dose=lifelong for all scenarios. HPV=human papillomavirus.

When assuming lower vaccine efficacy for one dose (one-dose efficacy=85%, two-dose efficacy=100%), 80% vaccination coverage at age 9 years and 70% at age 14 years, irrespective of the vaccination status, the model projected that a 5-year extended schedule would result in substantially less cervical cancer cases in the four countries than the current two-dose schedule (–5·9 to –7·5 percentage point difference in the relative reduction of cervical cancer incidence at equilibrium; figure 4, appendix 2 pp 6–13). This is because this strategy provides the opportunity to vaccinate girls who were missed when they were aged 9 years, and increase the percentage of girls with at least one dose. If vaccination coverage at age 14 years is 30%, irrespective of vaccination status, this 5-year extended strategy would still produce substantial reductions in cervical cancer cases, but would result in slightly more cervical cancers than the current two-dose schedule (3·2 to 4·6 percentage point difference in the relative reduction of cervical cancer incidence at equilibrium; figure 4).

**Figure 5:**
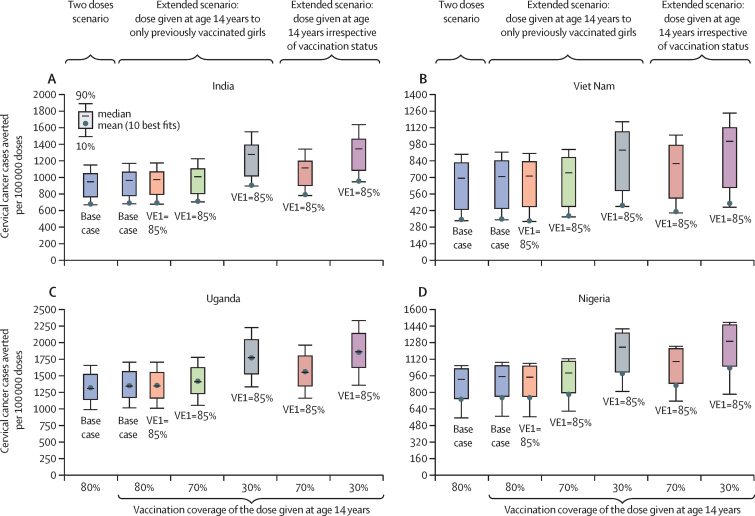
Projected efficiency of the current two-dose schedule and 5-year extended two-dose schedule, varying one-dose vaccine efficacy, and vaccination coverage at age 14 years, in India (A), Viet Nam (B), Uganda (C), and Nigeria (D) Projections are the mean of the ten best fitting parameter sets to the incidence of cervical cancer from the Global Cancer Observatory 2020. Uncertainty intervals should not be interpreted as confidence interval from a statistical point of view. Uncertainty intervals reflect uncertainty in model parameters and variability in HPV epidemiology within a country. Base case=vaccine efficacy at 100% and duration of protection is lifelong. VE=vaccine efficacy. VC=vaccination coverage. HPV=human papillomavirus.

Efficiency of vaccination (ie, the number of cancers averted per 100 000 doses given) was greater for the 5-year extended schedule than the current two-dose schedule, even with a drop in vaccination coverage for the second dose (figure 5, appendix 2 pp 6–13). Under base-case assumptions for the current two-dose schedule (two-dose efficacy=100%, duration=lifelong), the model projected that vaccinating 80% of girls aged 9 years would avert 671 cancer cases per 100 000 doses in India, 333 cancer cases per 100 000 doses in Viet Nam, 1305 cancer cases per 100 000 doses in Uganda, and 729 cancer cases per 100 000 doses in Nigeria. In contrast, a 5-year extended schedule with a lower efficacy for one dose (efficacy=85%, duration=lifelong) and with a second dose vaccination coverage of 70% would avert 706 cancer cases per 100 000 doses in India, 366 cancer cases per 100 000 doses in Viet Nam, 1406 cancer cases per 100 000 doses in Uganda, and 778 cancer cases per 100 000 doses in Nigeria; and with a second dose vaccination coverage of 30% would avert 899 cancer cases per 100 000 doses in India, 454 cancer cases per 100 000 doses in Viet Nam, 1756 cancer cases per 100 000 doses in Uganda, and 977 cancer cases per 100 000 doses in Nigeria (figure 5). Efficiency was greater for the 5-year extended schedule with a lower second dose vaccination coverage because the incremental impact of providing the first dose with 85% vaccine efficacy (*vs* no vaccination) is estimated to be substantially greater than the incremental impact of the second dose with 100% vaccine efficacy (*vs* the first dose). In other words, when the efficacy of the first dose is high (≥85%), giving the second dose does not provide substantial additional benefits.

When assuming a shorter duration of vaccine protection with one dose (one-dose duration=20 years, two-dose duration=lifelong) and 70% vaccination coverage for the second dose at age 14 years, the model projected slightly more cervical cancer cases than the current two-dose schedule assuming 80% vaccination coverage for both doses (2·1 to 7·6 percentage point difference in cervical cancer incidence at equilibrium; appendix 2 pp 6–14). If the vaccination coverage is 30% for the second dose at age 14 years, the model projected substantially more cervical cancer cases than the current two-dose schedule (15·3 to 35·5 percentage point difference in the relative reduction of cervical cancer incidence at equilibrium; appendix 2 p 14). The effect of a shorter duration of vaccine protection after one dose on the population-level impact of 5-year extended schedules is reduced if girls are vaccinated at age 14 years irrespective of their vaccination status. With 70% vaccination coverage of girls at age 14 years, irrespective of their vaccination status, the model projected differences of –2·8 to 8·5 percentage points in the relative reduction of cervical cancer incidence at equilibrium compared with the current two-dose schedule (appendix 2 pp 6–13, 15). With 30% coverage, the projected differences ranged from 13·0 to 33·5 percentage points. Finally, when assuming shorter vaccine duration of protection, the model projected that the efficiency of the 5-year extended schedules would be close to or higher than the current two-dose schedule, in both high (70%) and low (30%) vaccination coverage scenarios, and either vaccinating previously vaccinated girls or irrespective of vaccination status (appendix 2 pp 6–13, 16).

When assuming 65% coverage for the first and second doses in the current two-dose schedule, the model projected more cervical cancer cases than the current two-dose schedule at 80% vaccination coverage (13·4 to 16·6 percentage point difference in the relative reduction of cervical cancer incidence at equilibrium; appendix 2 pp 6–13, 17). However, when assuming 65% coverage for the dose at age 9 years and 65% or 50% coverage for the dose at age 14 years, given irrespective of the previous vaccination status, the model projected an impact closer to the current two-dose schedule at 80% vaccination coverage (–0·9 to 0·8 percentage point difference at equilibrium when vaccination coverage at age 14 years=65%, and 4·8 to 7·7 percentage point difference at equilibrium when vaccination coverage at age 14 years=50%; appendix 2 pp 6–13, 17). As previously observed, a shorter duration of protection (one-dose protection=20 years) had a greater effect on projections (appendix 2 pp 6–13, 18).

## Discussion

Our model projections suggest that the three main concerns regarding extended schedules should not substantially reduce the benefit projected with the current two-dose schedule. The first concern regarding girls becoming infected during the 5-year delay between the two doses should have a small effect on population-level impact. These results are driven by the following: (1) the proportion of girls becoming sexually active before age 15 years is relatively low in the four countries examined (estimated at 1% in Viet Nam to 19% in Nigeria, according to the Demographic and Health Surveys Program); (2) the prevalence of a high-risk HPV infection is also relatively low before age 14 years in those countries (estimated at <0·5% by our model fit; appendix 1), suggesting that sexual contact with an infected partner remains infrequent before age 14 years; (3) girls becoming sexually active and exposed to HPV in the 5-year delay between doses would be, at the very least, partially protected by their first dose. Results from a large multicentre prospective cohort study from India showed sustained high efficacy of a single dose of the vaccine against HPV16/18 persistent infection (95·4% (95% CI 85·0–99·9)) up to 10 years after vaccination.^13^

The second concern about lower coverage in older age groups depends on the drop in vaccination coverage. A small drop in vaccination coverage at age 14 years (eg, from 80% to 70%) would still produce very similar reductions in the number of cervical cancer cases prevented compared with the current two-dose schedule. However, a larger drop (eg, to 30%) could substantially reduce the number of cervical cancer cases prevented compared with the current two-dose schedule, especially if the one-dose duration of protection is short (eg, 20 years). However, a duration of protection of 20 years or less after one dose would be unlikely given that a recent study in India showed sustained protection of one dose through 10 years.^13^ We would have already observed a decline in protection if the average one-dose duration of protection was 20 years or less. Furthermore, in countries where school attendance drops substantially in girls aged 14 years, vaccinating girls at age 13 years could be considered (or the oldest age at which high-school attendance is achieved). Moreover, this drop assumes that only previously vaccinated girls are vaccinated at age 14 years, which is not necessarily the case if considering catch-up campaigns.

The third concern is that there could be implementation challenges of finding the girls to administer the second dose several years after the first dose. However, instead of finding these girls to provide a second dose, vaccinating girls at age 14 years, irrespective of their vaccination status, during routine catch-up campaigns could provide the opportunity to vaccinate girls who missed the first dose at age 9 years. By catching up girls at age 14 years and therefore increasing the number of girls vaccinated with at least one dose, our model projected that more cervical cancer cases would be prevented compared with the current two-dose schedule or the five-year extended schedule with a high coverage at age 14 years of previously vaccinated girls (appendix 2 pp 6–13).

Finally, an extended schedule might be easier to implement with WHO's 2022 recommendation that one or two-dose schedules could be considered for girls aged 9–14 years,^19^ because it could provide a security net for countries who are uncertain about starting directly with or switching to a one-dose strategy.

Our model projections also suggest that almost all extended scenarios examined are more efficient than the current two-dose schedule, even when assuming a lower efficacy of one dose, lower duration of protection of one dose, or lower vaccination coverage at age 14 years. If both the vaccine efficacy of the first dose and vaccination coverage for the second dose are lower for the extended schedules (vaccine efficacy of dose 1=85%, vaccination coverage of dose 2=70% to 30% among previously vaccinated girls) compared with the current two-dose schedule (vaccine efficacy of dose 1=100%, vaccination coverage of dose 2=80%), the number of cervical cancer cases prevented would be slightly reduced, but the efficiency per dose would be greater. Indeed, when the first dose is highly effective (eg, 85%), the additional benefit of providing a second dose is reduced.^7^ The efficiency is projected to be even greater if the dose at age 14 years is provided irrespective of vaccination status. For example, in India, 950 cervical cancer cases would be averted per 100 000 doses for the 5-year extended schedule with 30% coverage at age 14 years, irrespective of vaccination status (vaccine efficacy of dose 1=85%) compared with 671 cases averted per 100 000 doses for the current two-dose schedule. By providing the opportunity to vaccinate a greater number of girls with at least one dose (rather than giving two doses to a smaller number of girls), such a routine catch-up campaign vaccination strategy is projected to be highly efficient, particularly in the context of a highly efficacious first dose.^13^ The most efficient strategy is the one that maximises cases prevented per dose given, which is a key consideration when doses are scarce.

These results showing high population-level impact and efficiency of the two-dose extended schedules have important implications, particularly in the context of the COVID-19 pandemic. The pandemic has affected public health programmes worldwide. Significant decreases of vaccination coverage for recommended vaccines for children and adolescents, including the HPV vaccine, have been documented in several countries.^20^, ^21^ For example, a decrease of about 17 percentage points in HPV vaccination coverage in low-income countries, 11 percentage points in middle-income countries, and 10 percentage points in high-income countries was estimated in 2020 compared with 2019.^21^ Furthermore, several countries that had planned to introduce HPV vaccination were unable to do so because of the pandemic.^22^ When considering the disruptions caused by the COVID-19 pandemic, organising HPV routine vaccination with two doses in the same year might represent a major challenge. A 5-year extended dose schedule could offer an effective and efficient strategy to introduce or reintroduce HPV vaccination while providing a 5-year time window to reassess whether it is necessary to give the second dose and to improve access to cervical screening and treatment. Furthermore, in a previous analysis, we showed that a catch-up campaign vaccination at age 14 years starting at the same time as routine vaccination of girls aged 9 years could provide the opportunity to vaccinate girls with at least one dose just before they become older than 14 years (and thus prevent a substantial number of cervical cancer cases).^7^

To our knowledge, this is the first study to comprehensively examine the population-level impact and efficiency of a 5-year HPV vaccination extended dose schedule in LMICs, with varying one-dose efficacy and duration, and vaccination coverage for the second dose. Our study has major strengths. First, we used an individual-based transmission-dynamic model calibrated to LMIC-specific behavioural and epidemiological data from four countries (HPV-ADVISE LMIC). Second, despite differences in sexual activity and cervical cancer burden between the four LMICs included in this study, the population-level impact and efficiency of an extended vaccination schedule were consistent across the four countries. Our results are most likely generalisable to other LMICs with similar HPV and cervical cancer epidemiological profiles and should be considered as general principles guiding HPV vaccination policies in different countries. Third, our model projections were based on 1000 runs simulated from 50 parameter sets that capture uncertainty and variability in sexual behaviour, HPV transmission, and natural history of HPV-related diseases within a country.

Our study also has some limitations. First, although we modelled several scenarios of extended schedules by varying vaccine efficacy and duration of protection after one dose, and vaccination coverage at age 14 years, we did not model the complete range of potential scenarios (eg, lower vaccine efficacy). However, our results were robust to decreases in one-dose duration of protection (lifelong to 20 years) and efficacy (100% to 85%) and recent results suggested that one-dose efficacy should be higher than 85%.^13^ Second, we did not vary the 5-year interval between the two doses (ie, age at which the second dose is given). However, our results should be considered as general principles guiding decisions about HPV vaccination. The 5-year interval and age at second dose could be adapted to each country's context related to age at sexual debut or school drop-out. Third, data about the start of sexual activity are scarce in many countries, including the four LMICs modelled in this analysis. To take into consideration the uncertainty related to sexual activity data, we selected four LMICs with very different sexual activity profiles and used 50 different parameter sets for model projections for each country. Fourth, we present the mean of the ten best fitting parameter sets to the average country-specific cervical cancer incidence estimates from 2020 from the Global Cancer Observatory. However, cervical cancer incidence might be underestimated if cervical cancer cases are under-reported in some countries or some regions within a country. We used the 50 parameter sets identified through calibration using several data sources to show the potential variability in cervical cancer incidence within a country (appendix 1 p 8). For example, there is substantial variability in cervical cancer incidence between regions in India and Viet Nam.^16^, ^17^ However, although there is variability in the baseline cervical cancer incidence, our conclusions remain consistent for different prevaccination cervical cancer incidence.

In summary, a 5-year extended HPV vaccination schedule represents an effective and efficient HPV vaccination strategy, particularly in the context of HPV vaccine supply shortage and scarce human and financial resources, which has been exacerbated by the COVID-19 pandemic in many countries. This strategy could allow countries to gradually introduce or reintroduce HPV vaccination while providing a 5-year window to reassess the necessity of giving a second dose.

## Data sharing

No individual participant-level data were used in this study. Descriptions of the model structure, the parameters included in the model, and the empirical data used for calibration and validation are available in appendix 1 (https://marc-brisson.net/HPVadvise-LMIC.pdf).

## Declaration of interests

MB, MD, and MJ are members of the Single-Dose HPV Vaccine Evaluation Consortium. EB, J-FL, KP, and M-CB declare no competing interests.
