# Supplementary material for: Potential benefit of extended dose schedules of human papillomavirus vaccination in the context of scarce resources and COVID-19 disruptions in low-income and middle-income countries: a mathematical modelling analysis
Source: Lancet Glob Health. 2022 Dec 13;11(1):e48–58. doi: 10.1016/S2214-109X(22)00475-2 (PMC9764452; doi:10.1016/S2214-109X(22)00475-2)
Supplement: Supplementary appendix 2 [file mmc2.pdf]

THE LANCET
Global Health

Supplementary appendix 2

This appendix formed part of the original submission and has been peer reviewed. We post it as supplied by the authors.

Supplement to: Bénard E, Drolet M, Laprise J-F, et al. Potential benefit of extended dose schedules of human papillomavirus vaccination in the context of scarce resources and COVID-19 disruptions in low-income and middle-income countries: a mathematical modelling analysis. *Lancet Glob Health* 2023; **11:** e48–58.

Supplementary appendix

Supplement to: Bénard É, Drolet M, Laprise J-F, Jit M, Prem K, Boily M-C, Brisson M. Potential benefit of extended dose schedules of human papillomavirus (HPV) vaccination in the context of limited resources and COVID-19 disruptions in low- and middle-income countries: A mathematical modeling analysis.

Supplementary Appendix

Table S1. Sexual behaviour and cervical cancer incidence in India, Vietnam, Uganda and Nigeria........................3

Table S2. HPV-FRAME...4

Table S3. Relative cervical cancer incidence reduction (RR), cases averted and cases averted per 100,000 doses in India, Vietnam, Uganda and Nigeria..6

Figure S1. Projected population-level impact of five-year extended two-dose schedule, with one-dose duration of protection of 20 years and second dose given to previously vaccinated girls only 14

Figure S2. Projected population-level impact of five-year extended two-dose schedule, with one-dose duration of protection of 20 years and second dose given at 14 years old, irrespective of vaccination status. 15

Figure S3. Projected efficiency of the current two-dose schedule and five-year extended two-dose schedule with duration of one-dose protection of 20 years. 16

Figure S4. Projected population-level impact of five-year extended two-dose schedule, assuming 65% vaccination coverage at 9 years old, one-dose vaccine efficacy of 85% and second dose given at 14 years old, irrespective of vaccination status...17

Figure S5. Projected population-level impact of five-year extended two-dose schedule, assuming 65% vaccination coverage at 9 years old, one-dose duration of protection of 20 years and second dose given at 14 years old, irrespective of vaccination status. 18

Figure S6. Projected efficiency of the current two-dose schedule and five-year extended two-dose schedule, assuming 65% vaccination coverage at 9 years old...19

Estimation of country-specific population size between 2100-2120...20

Table S1. Sexual behaviour and cervical cancer incidence in India, Vietnam, Uganda and Nigeria[1-3]

	India	Vietnam	Uganda	Nigeria
**Sexual behaviour[1-2]**				
Mean number of lifetime partners				
Women	1.7	1.0	2.3	1.5
Men	1.9	1.4	6.3	4.1
Had sex by 15 years old (proportion, %)	9.9	0.7	20.0	23.7
Partner > 10 years older (for 15-19 year-old girls) (proportion, %)	10.6	6.3	10.0	39.4
**Cervical cancer incidence by age (per 100,000 women-year)[3]**				
15-39 years old	5.6	3.4	25.7	4.7
40-44 years old	26.7	12.3	98.1	35.3
45-49 years old	35.8	16.5	117.5	53.1
50-54 years old	42.1	20.2	134.6	74.1
55-59 years old	44.8	22.3	152.5	104.2
60-64 years old	45.9	23.1	165.2	126.7
65-69 years old	46.8	22.6	164.4	127.2
70-74 years old	47.4	21.4	162.4	112.8
75+	48.8	17.7	166.5	65.6

Ref: . 1. DHS Program. The Demographic and health surveys program 2013 and 2016. Available at http://dhsprogram.com/. Accessed June 21, 2022. 2. Multiple Indicator Survey. UNICEF. Available at: https://mics.unicef.org/tools#reporting. Accessed June 8, 2022. 3. WHO / International Agency for research on Cancer. Global Cancer Observatory 2020: Estimated Cancer Incidence, Mortality and Prevalence Worldwide in 2020-Cervical Cancer. (available at: https://gco.iarc.fr/today/home). Accessed June 8, 2022.

Table S2. HPV-FRAME[4]

A. Inputs	Reported by age? (Y/N)	Report by sex?	Comments
**Core reporting standard**			
Target population for intervention	Y	Y	Vaccination of girls at 9 and 14 years old (see section Methods of the manuscript).
Sexual behavior	Y	Y	Sexual behavior inputs used (number of lifetime partners, number of partners in the last year, partnership formation, etc) are described in the Technical Appendix, section 2.2 (https://marc-brisson.net/HPVadvise-LMIC.pdf).
Cohort examined for evaluation/ time horizon	Y (multiple cohorts)	Y (multiple cohorts)	100 year time horizon from start of vaccination (2021-2120). Intervention is given to cohorts, but we examine the outcome in the population.
Quality of life assumptions	Not applicable	Not applicable	This study focuses on the impact of vaccination on health outcomes only (cervical cancer).
Calibration	Y	Y	HPV-ADVISE LMIC was calibrated with country-specific behavioral and epidemiological data (see Technical Appendix, section 2 - https://marc-brisson.net/HPVadvise-LMIC.pdf).
Validation (where possible)	Y	Y	HPV-ADVISE LMIC has been validated and previously used to model various HPV vaccination and cervical cancer screening strategies (see Technical Appendix, section 2.4 for details on validation - https://marc-brisson.net/HPVadvise-LMIC.pdf).
Costs	Not applicable	Not applicable	This study focuses on the impact of vaccination on health outcomes only (cervical cancer).
**Reporting standards for models of vaccination in adolescent individuals**			
Vaccine coverage	Y	Y	Intervention is for girls only. See section Methods of the manuscript for specific coverage.
Vaccine efficacy	Y	Y	See section Methods of the manuscript for specific efficacy.
Vaccine cross-protection	Not applicable	Not applicable	The study focuses on the nonavalent vaccine, which includes HPV16, 18, 31, 33, 45, 52 and 58.
Duration vaccine protection and waning	Y	Y	See section Methods of the manuscript for specific duration of protection.
Vaccine and delivery costs	Not applicable	Not applicable	This study focuses on the impact of vaccination on health outcomes only (cervical cancer).
Pre-vaccination disease burden	Y	Y	Pre-vaccination epidemiological data were used for calibration (see Technical Appendix, section 2.3 - https://marc-brisson.net/HPVadvise-LMIC.pdf).
Duration of natural immunity	Y	Y	See Technical Appendix, section 2.2 (https://marc-brisson.net/HPVadvise-LMIC.pdf).

**Reporting standards for evaluations assessing alternative vaccine types or reduced-dose schedules**			
Timing between doses	Y	Y	5-year extended vaccination schedule (see section Methods of the manuscript)
**Reporting standards for models of HPV prevention in LMIC**			
HIV prevalence rates, if endemic in country	N	N	We did not account for HIV prevalence (see section Discussion of the manuscript).
Description of any opportunistic or pilot/demonstration screening projects ongoing	N	N	See Technical Appendix, section 2.2 for screening modeling (https://marc-brisson.net/HPVadvise-LMIC.pdf).

**B. Outputs**	**Reported by age? (Y/N)**	**Report by sex?**	**Comments**
**Core reporting standards**			
Cancer incidence, mortality, life years, QALYs/DALYs (as appropriate)	Y	Y	We present the absolute number of cervical cancer cases prevented per 100,000 vaccine doses and the relative reduction in cervical cancer incidence (see section Results of the manuscript).
HPV prevalence, pre-intervention	N	N	Pre-intervention prevalence is used for calibration and is reported in the Technical Appendix, section 2.3 (https://marc-brisson.net/HPVadvise-LMIC.pdf).
CIN2/3 detected	N	N	This outcome is not reported as the present study focuses on the impact of vaccination on cervical cancer.
Sensitivity analysis on key inputs	Y	Y	See sections Methods and Results of the manuscript.
Incremental cost-effectiveness ratios and costs saved	Not applicable	Not applicable	This study focuses on the impact of vaccination on health outcomes only (cervical cancer).
**Reporting standards for models of vaccination in adolescent individuals**			
Absolute reductions in HPV infections, and/or warts, post-vaccination	N	N	This study focuses on the impact of vaccination on cervical cancer.Previous papers have shown dynamic for HPV infection over time.[5]
Absolute reductions in CIN2+ post-vaccination	N	N	This study focuses on the impact of vaccination on cervical cancer.
Absolute reductions in invasive cancer (cervical and other HPV cancers, as relevant) post-vaccination	Y	Y	We present the absolute number of cervical cancer cases prevented per 100,000 vaccine doses and the relative reduction in cervical cancer incidence (see section Results of the manuscript).

Ref: 4.Canfell K, Kim JJ, Kulasingam S, Berkhof J, Barnabas R, Bogaards JA, et al. HPV-FRAME: A consensus statement and quality framework for modelled evaluations of HPV-related cancer control. *Papillomavirus Res*. 2019;8:100184. 5. Drolet M, Laprise JF, Martin D, et al. Optimal human papillomavirus (HPV) vaccination strategies to prevent cervical cancer in low- and middle-income countries in the context of limited resources: A mathematical modeling analysis. *Lancet Infect Dis*. 2021;21(11):1598-1610.

Table S3. Relative cervical cancer incidence reduction (RR), cases averted and cases averted per 100,000 doses in India, Vietnam, Uganda and Nigeria

Vaccination scenarios	Proportion of girls vaccinated with ≥ 1 dose	1 dose only	2 doses	RR of cervical cancer incidence at equilibrium Mean (80% UI+)	Percentage point difference vs current 2-dose Mean (80% UI)	Cases averted (millions) Mean (80% UI)	Difference in cases averted vs current 2-dose (millions) Mean (80% UI)	Total number of doses (millions)	Cases averted/ 100 000 doses Mean (80% UI)
**INDIA**									
**A. Current 2-dose schedule (2 doses at 9 yrs old)**									
Coverage at 9 yrs old: 80%	80%	0%	80%	85.2% (77.0% to 89.1%)	Ref	9.68 (9.68* to 16.65)	Ref	1,443	671 (671 to 1,153)
**Extended schedules (1 dose at 9 yrs old and 1 dose at 14yrs old)**									
B1. According to coverage at 14 yrs old among previously vaccinated girls, (VE_1=100%, VD_1=lifelong, VC_1=80%)									
Coverage at 14 yrs (previously vaccinated girls only):									
80%	80%	0%	80%	85.2% (77.0% to 89.1%)	0.0 (0.0 to 0.0)	9.68 (9.68* to 16.65)	0 (0 to 0)	1,417	684 (684 to 1,174)
70%	80%	0%	70%	85.2% (77.0% to 89.1%)	0.0 (0.0 to 0.0)	9.68 (9.68* to 16.65)	0 (0 to 0)	1,330	728 (728 to 1,252)
30%	80%	0%	30%	85.2% (77.0% to 89.1%)	0.0 (0.0 to 0.0)	9.68 (9.68* to 16.65)	0 (0 to 0)	982	986 (986 to 1,695)
B2. According to coverage at 14 yrs old among previously vaccinated girls and lower 1-dose efficacy (VE_1=85%, VD_1=Lifelong, VC_1=80%)									
Coverage at 14 yrs (previously vaccinated girls only):									
80%	80%	0%	80%	84.7% (76.7% to 88.9%)	0.5 (-2.8 to 3.3)	9.71 (9.71* to 16.71)	0.03 (-0.25 to 0.29)	1,417	686 (686 to 1,179)
70%	80%	0%	70%	82.8% (73.3% to 87.1%)	2.4 (-1.4 to 5.0)	9.39 (9.39* to 16.35)	-0.29 (-0.52 to 0.12)	1,330	706 (706 to 1,229)
30%	80%	0%	30%	78.3% (70.1% to 82.4%)	6.9 (3.8 to 11.3)	8.83 (8.83* to 15.29)	-0.86 (-1.57 to -0.84)	982	899 (899 to 1,556)
C. According to coverage at 14 yrs old irrespective of vaccination status and lower 1-dose efficacy (VE^1=85%, VD^1=Lifelong, VC^1=80%)									
Coverage at 14 yrs (irrespective of vaccination status):									
70%	94%	38%	56%	91.9% (82.6% to 94.9%)	-6.7 (-9.0 to -3.7)	10.41 (10.41* to 17.93)	0.73 (0.63 to 1.28)	1,330	783 (783 to 1,346)
30%	86%	62%	24%	81.7% (74.1% to 86.3%)	3.5 (0.4 to 6.3)	9.33 (9.33* to 16.15)	-0.35 (-0.91 to -0.35*)	982	950 (950 to 1,643)

Vaccination scenarios	Proportion of girls vaccinated with ≥ 1 dose	1 dose only	2 doses	RR of cervical cancer incidence at equilibrium Mean (80% UI+)	Percentage point difference vs current 2-dose Mean (80% UI)	Cases averted (millions) Mean (80% UI)	Difference in cases averted vs current 2-dose (millions) Mean (80% UI)	Total number of doses (millions)	Cases averted/ 100 000 doses Mean (80% UI)
**Sensitivity analysis for extended schedules with shorter duration of 1-dose protection** (VE_1=100%, VD_1=20 yrs, VC_1=80%)									
Coverage at 14yrs among previously vaccinated girls only:									
80%	80%	0%	80%	85.2% (77.0% to 89.1%)	0.0 (0.0 to 0.0)	9.68 (9.68* to 16.65)	0 (0 to 0)	1,417	684 (684 to 1,174)
70%	80%	0%	70%	81.1% (72.8% to 85.5%)	4.1 (0.4 to 7.8)	9.25 (9.25* to 15.95)	-0.43 (-0.96 to -0.36)	1,330	696 (688 to 1,199)
30%	80%	0%	30%	64.4% (52.7% to 70.9%)	20.8 (16.5 to 26.2)	7.08 (7.06 to 12.13)	-2.60 (-4.87 to -2.54)	982	721 (693 to 1,233)
Coverage at 14yrs among irrespective of vaccination status:									
70%	94%	38%	56%	83.8% (73.8% to 88.7%)	1.5 (-2.1 to 3.9)	9.54 (9.54* to 16.38)	-0.14 (-0.83 to 0.0007)	1,330	717 (711 to 1,231)
30%	86%	62%	24%	65.2% (54.2% to 71.9%)	20.0 (15.7 to 25.9)	7.14 (7.11 to 12.21)	-2.55 (-4.72 to -2.54)	982	727 (709 to 1,243)
**Sensitivity analysis for extended schedules with lower coverage at 9 years old (VC_1=65%)**									
Current 2-dose schedule (2 doses at 9 years old, VC=65%)	65%	0%	65%	71.8% (64.3% to 75.1%)	13.4 (10.1 to 17.5)	7.99 (7.99* to 13.81)	-1.69 (-2.90 to -1.69*)	1,172	682 (680 to 1,177)
Coverage at 14yrs among irrespective of vaccination status:									
65% (VE_1=85%)	88%	46%	42%	84.4% (77.3% to 88.3%)	0.8 (-2.4 to 3.0)	9.55 (9.55* to 16.61)	-0.13 (-0.41 to 0.14)	1,151	830 (830 to 1,442)
50% (VE_1=85%)	83%	50%	33%	77.6% (68.7% to 83.7%)	7.7 (2.8 to 11.0)	9.01 (9.01* to 15.59)	-0.67 (-1.33 to -0.67*)	1,021	883 (883 to 1,526)
65% (VD_1=20 yrs)	88%	46%	42%	82.9% (73.8% to 86.6%)	2.3 (-0.4 to 6.8)	9.315 (9.31* to 16.01)	-0.38 (-1.05 to -0.24)	1,151	808 (807 to 1,388)
50% (VD_1=20 yrs)	83%	50%	33%	73.7% (63.9% to 80.0%)	11.5 (8.3 to 16.1)	8.16 (8.16* to 14.24)	-1.52 (-2.70 to -1.49)	1,021	800 (800 to 1,392)

	Proportion of girls vaccinated with			RR of cervical cancer incidence at equilibrium	Percentage point difference vs current 2-dose	Cases averted (millions)	Difference in cases averted vs current 2-dose (millions)	Total number of doses (millions)	Cases averted/ 100 000 doses
Vaccination scenarios	≥ 1 dose	1 dose only	2 doses	Mean (80% UI[+])	Mean (80% UI)	Mean (80% UI)	Mean (80% UI)		Mean (80% UI)
**VIETNAM**									
**A. Current 2-dose schedule (2 doses at 9yrs old)**									
Coverage at 9yrs old: 80%	80%	0%	80%	85.8% (78.9% to 86.9%)	Ref	0.32 (0.32* to 0.86)	Ref	96	333 (333 to 896)
**Extended schedules (1 dose at 9 yrs old and 1 dose at 14yrs old**									
B1. According to coverage at 14 yrs old among previously vaccinated girls, (VE_1=100%, VD_1=lifelong, VC_1=80%)									
Coverage at 14 yrs (previously vaccinated girls only):									
80%	80%	0%	80%	85.8% (78.9% to 86.9%)	0.0 (0.0 to 0.0)	0.32 (0.32* to 0.86)	0 (0 to 0)	94	340 (340 to 915)
70%	80%	0%	70%	85.8% (78.9% to 86.9%)	0.0 (0.0 to 0.0)	0.32 (0.32* to 0.86)	0 (0 to 0)	88	362 (362 to 974)
30%	80%	0%	30%	85.8% (78.9% to 86.9%)	0.0 (0.0 to 0.0)	0.32 (0.32* to 0.86)	0 (0 to 0)	65	490 (490 to 1,318)
B2. According to coverage at 14 yrs old among previously vaccinated girls and lower 1-dose efficacy (VE_1=85%, VD_1=Lifelong, VC_1=80%)									
Coverage at 14yrs (previously vaccinated girls only):									
80%	80%	0%	80%	83.8% (79.9% to 86.0%)	2.0 (-3.2 to 2.8)	0.31 (0.31* to 0.85)	-0.01 (-0.03 to 0.03)	94	327 (327 to 903)
70%	80%	0%	70%	84.2% (77.8% to 85.2%)	1.5 (-2.1 to 4.0)	0.32 (0.32* to 0.83)	0.003 (-0.04 to 0.01)	88	366 (366 to 937)
30%	80%	0%	30%	78.3% (71.2% to 79.4%)	7.5 (4.2 to 10.7)	0.30 (0.30* to 0.77)	-0.02 (-0.09 to -0.003)	65	454 (454 to 1,172)
C. According to coverage at 14 yrs old irrespective of vaccination status and lower 1-dose efficacy (VE_1=85%, VD_1=Lifelong, VC_1=80%)									
Coverage at 14 yrs (irrespective of vaccination status):									
70%	94%	38%	56%	93.3% (87.2% to 94.1%)	-7.5 (-11.3 to -4.0)	0.36 (0.36* to 0.94)	0.04 (0.03 to 0.09)	88	402 (399 to 1,059)
30%	86%	62%	24%	81.1% (74.8% to 83.3%)	4.6 (-1.5 to 9.3)	0.31 (0.31* to 0.82)	-0.01 (-0.06 to 0.01)	65	472 (447 to 1,245)

Vaccination scenarios	Proportion of girls vaccinated with ≥ 1 dose	1 dose only	2 doses	RR of cervical cancer incidence at equilibrium Mean (80% UI[+])	Percentage point difference vs current 2-dose Mean (80% UI)	Cases averted (millions) Mean (80% UI)	Difference in cases averted vs current 2-dose (millions) Mean (80% UI)	Total number of doses (millions)	Cases averted/ 100 000 doses Mean (80% UI)
**Sensitivity analysis for extended schedules with shorter duration of 1-dose protection** (VE_1=100%, VD_1=20 yrs, VC_1=80%)									
Coverage at 14yrs among previously vaccinated girls only:									
80%	80%	0%	80%	85.8% (78.9% to 86.9%)	0.0 (0.0 to 0.0)	0.32 (0.32* to 0.86)	0 (0 to 0)	94	340 (340 to 915)
70%	80%	0%	70%	78.2% (72.4% to 80.7%)	7.6 (2.5 to 9.7)	0.30 (0.30* to 0.79)	-0.03 (-0.08 to -0.01)	88	333 (333 to 895)
30%	80%	0%	30%	50.3% (47.4% to 57.9%)	35.5 (24.5 to 35.7)	0.19 (0.19 to 0.54)	-0.13 (-0.34 to -0.13*)	65	295 (285 to 817)
Coverage at 14yrs among irrespective of vaccination status:									
70%	94%	38%	36%	77.3% (73.4% to 82.7%)	8.5 (1.1 to 10.0)	0.30 (0.30 to 0.77)	-0.02 (-0.09 to -0.01)	88	341 (325 to 867)
30%	86%	62%	24%	52.3% (48.7% to 57.8%)	33.5 (25.0 to 35.0)	0.178 (0.178* to 0.51)	-0.14 (-0.35 to -0.13)	65	271 (248 to 772)
**Sensitivity analysis for extended schedules with lower coverage at 9 years old (VC_1=65%)**									
Current 2-dose schedule (2 doses at 9 years old, VC=65%)	65%	0%	65%	69.2% (61.6% to 71.7%)	16.6 (10.7 to 20.1)	0.27 (0.27* to 0.70)	-0.06 (-0.15 to -0.04)	78	339 (328 to 884)
Coverage at 14yrs among irrespective of vaccination status:									
65% (VE_1=85%)	88%	46%	42%	85.3% (79.3% to 86.7%)	0.5 (-4.0 to 3.9)	0.33 (0.33* to 0.86)	0.008 (-0.02 to 0.03)	77	429 (426 to 1,120)
50% (VE_1=85%)	83%	50%	33%	79.1% (73.9% to 80.8%)	6.7 (1.2 to 8.0)	0.31 (0.31* to 0.80)	-0.009 (-0.07 to -0.0008)	68	459 (452 to 1,171)
65% (VD_1=20 yrs)	88%	46%	42%	77.6% (73.3% to 80.7%)	8.2 (1.5 to 9.2)	0.30 (0.30 to 0.79)	-0.02 (-0.07 to -0.01)	77	387 (382 to 1,025)
50% (VD_1=20 yrs)	83%	50%	33%	67.9% (62.1% to 70.7%)	17.8 (11.8 to 20.4)	0.26 (0.25 to 0.68)	-0.06 (-0.18 to -0.06*)	68	377 (372 to 1,001)

Vaccination scenarios	Proportion of girls vaccinated with ≥ 1 dose	1 dose only	2 doses	RR of cervical cancer incidence at equilibrium Mean (80% UI[+])	Percentage point difference vs current 2-dose Mean (80% UI)	Cases averted (millions) Mean (80% UI)	Difference in cases averted vs current 2-dose (millions) Mean (80% UI)	Total number of doses (millions)	Cases averted/ 100 000 doses Mean (80% UI)
**UGANDA**									
**A. Current 2-dose schedule (2 doses at 9yrs old)**									
Coverage at 9yrs old: 80%	80%	0%	80%	80.2% (75.8% to 84.7%)	Ref	2.82 (2.13 to 3.59)	Ref	216	1,305 (986 to 1,657)
**Extended schedules (1 dose at 9yrs-old and 1 dose at 14yrs-old)**									
B1. According to coverage at 14 yrs old among previously vaccinated girls, (VE_1=100%, VD_1=lifelong, VC_1=80%)									
Coverage at 14yrs (previously vaccinated girls only):									
80%	80%	0%	80%	80.2% (75.8% to 84.7%)	0.0 (0.0 to 0.0)	2.82 (2.13 to 3.59)	0 (0 to 0)	211	1,341 (1,014 to 1,704)
70%	80%	0%	70%	80.2% (75.8% to 84.7%)	0.0 (0.0 to 0.0)	2.82 (2.13 to 3.59)	0 (0 to 0)	198	1,428 (1,080 to 1,816)
30%	80%	0%	30%	80.2% (75.8% to 84.7%)	0.0 (0.0 to 0.0)	2.82 (2.13 to 3.59)	0 (0 to 0)	147	1,927 (1,456 to 2,450)
B2. According to coverage at 14 yrs old among previously vaccinated girls and lower 1-dose efficacy (VE_1=85%, VD_1=Lifelong, VC_1=80%)									
Coverage at 14yrs (previously vaccinated girls only):									
80%	80%	0%	80%	79.2% (74.2% to 84.8%)	1.0 (-2.0 to 3.1)	2.82 (2.16 to 3.59)	-0.004 (-0.03 to 0.01)	211	1,340 (1,008 to 1,705)
70%	80%	0%	70%	78.1% (74.4% to 83.3%)	2.1 (-0.3 to 3.6)	2.78 (2.09 to 3.52)	-0.04 (-0.08 to -0.03)	198	1,406 (1,052 to 1,777)
30%	80%	0%	30%	72.6% (66.6% to 78.1%)	7.6 (4.8 to 10.2)	2.57 (1.96 to 3.27)	-0.25 (-0.32 to -0.19)	147	1,756 (1,332 to 2,229)
C. According to coverage at 14 yrs old irrespective of vaccination status and lower 1-dose efficacy (VE_1=85%, VD_1=Lifelong, VC_1=80%)									
Coverage at 14 yrs (irrespective of vaccination status):									
70%	94%	38%	56%	86.1% (81.0% to 90.9%)	-5.9 (-8.5 to -4.2)	3.06 (2.30 to 3.88)	0.24 (0.17 to 0.29)	198	1,548 (1,160 to 1,962)
30%	86%	62%	24%	77.0% (71.9% to 82.1%)	3.2 (0.8 to 5.6)	2.70 (2.04 to 3.42)	-0.12 (-0.16 to 0.08)	147	1,845 (1,357 to 2,335)

Vaccination scenarios	Proportion of girls vaccinated with ≥ 1 dose	1 dose only	2 doses	RR of cervical cancer incidence at equilibrium Mean (80% UI[+])	Percentage point difference vs current 2-dose Mean (80% UI)	Cases averted (millions) Mean (80% UI)	Difference in cases averted vs current 2-dose (millions) Mean (80% UI)	Total number of doses (millions)	Cases averted/ 100 000 doses Mean (80% UI)
**Sensitivity analysis for extended schedules with shorter duration of 1-dose protection** (VE$_1$=100%, VD$_1$=20 yrs, VC$_1$=80%)									
Coverage at 14yrs among previously vaccinated girls only:									
80%	80%	0%	80%	80.2% (75.8% to 84.7%)	0.0 (0.0 to 0.0)	2.82 (2.13 to 3.59)	0 (0 to 0)	211	1,341 (1,014 to 1,704)
70%	80%	0%	70%	76.0% (71.4% to 81.7%)	4.2 (1.7 to 6.1)	2.70 (2.05 to 3.42)	-0.13 (-0.16 to -0.08)	198	1,363 (1,035 to1,728)
30%	80%	0%	30%	60.6% (54.7% to 65.2%)	19.6 (15.9 to 25.1)	2.12 (1.64 to 2.72)	-0.70 (-0.93 to -0.49)	147	1,447 (1,031 to 1,847)
Coverage at 14yrs among irrespective of vaccination status:									
70%	94%	38%	36%	79.2% (75.1% to 83.9%)	1.0 (-2.1 to 3.2)	2.81 (2.14 to 3.56)	-0.01 (-0.07 to 0.06)	198	1,423 (1,057 to 1,796)
30%	86%	62%	24%	61.2% (56.8% to 66.8%)	19.0 (13.3 to 23.2)	2.16 (1.68 to 2.78)	-0.66 (-0.88 to -0.44)	147	1,475 (1,102 to 1,891)
**Sensitivity analysis for extended schedules with lower coverage at 9 years old (VC$_1$=65%)**									
Current 2-dose schedule (2 doses at 9 years old, VC=65%)	65%	0%	65%	66.3% (62.2% to 71.5%)	13.9 (11.7 to 15.9)	2.35 (1.78 to 3.00)	-0.48 (-0.60 to -0.38)	176	1,334 (999 to 1,706)
Coverage at 14yrs among irrespective of vaccination status:									
65% (VE$_1$=85%)	88%	46%	42%	79.4% (74.3% to 84.8%)	0.8 (-2.0 to 2.7)	2.83 (2.13 to 3.59)	0.003 (-0.02 to 0.03)	171	1,653 (1,246 to 2,100)
50% (VE$_1$=85%)	83%	50%	33%	74.4% (71.1% to 80.1%)	5.8 (2.8 to 7.5)	2.64 (2.00 to 3.34)	-0.18 (-0.24 to -0.13)	152	1,741 (1,314 to 2,200)
65% (VD$_1$=20 yrs)	88%	46%	42%	77.7% (74.4% to 83.1%)	2.5 (-0.8 to 4.6)	2.76 (2.08 to 3.50)	-0.07 (-0.11 to -0.01)	171	1,612 (1,203 to 2,041)
50% (VD$_1$=20 yrs)	83%	50%	33%	69.5% (65.2% to 74.8%)	10.7 (7.3 to 14.0)	2.45 (1.86 to 3.10)	-0.38 (-0.49 to -0.26)	152	1,610 (1,182 to 2,036)

Vaccination scenarios	Proportion of girls vaccinated with: ≥ 1 dose	1 dose only	2 doses	RR of cervical cancer incidence at equilibrium<br>Mean (80% UI[+])	Percentage point difference vs current 2-dose<br>Mean (80% UI)	Cases averted (millions)<br>Mean (80% UI)	Difference in cases averted vs current 2-dose (millions)<br>Mean (80% UI)	Total number of doses (millions)	Cases averted/ 100 000 doses<br>Mean (80% UI)
**NIGERIA**									
**A. Current 2-dose schedule (2 doses at 9yrs old)**									
Coverage at 9yrs old: 80%	80%	0%	80%	78.6%<br>(62.5% to 84.3%)	Ref	6.02<br>(4.96 to 8.75)	Ref	826	729<br>(555 to 1,060)
**Extended schedules (1 dose at 9yrs-old and 1 dose at 14yrs-old)**									
B1. According to coverage at 14 yrs old among previously vaccinated girls, (VE_1=100%, VD_1=lifelong, VC_1=80%)									
Coverage at 14yrs (previously vaccinated girls only):									
80%	80%	0%	80%	78.6%<br>(62.5% to 84.3%)	0.0<br>(0.0 to 0.0)	6.02<br>(4.96 to 8.75)	0<br>(0 to 0)	802	750<br>(572 to 1,091)
70%	80%	0%	70%	78.6%<br>(62.5% to 84.3%)	0.0<br>(0.0 to 0.0)	6.02<br>(4.96 to 8.75)	0<br>(0 to 0)	754	798<br>(658 to 1,161)
30%	80%	0%	30%	78.6%<br>(62.5% to 84.3%)	0.0<br>(0.0 to 0.0)	6.02<br>(4.96 to 8.75)	0<br>(0 to 0)	559	1,076<br>(887 to 1,566)
B2. According to coverage at 14 yrs old among previously vaccinated girls and lower 1-dose efficacy (VE_1=85%, VD_1=Lifelong, VC_1=80%)									
Coverage at 14yrs (previously vaccinated girls only):									
80%	80%	0%	80%	78.3%<br>(61.6% to 84.5%)	0.3<br>(-2.5 to 2.8)	5.99<br>(4.94 to 8.68)	-0.03<br>(-0.10 to 0.06)	802	746<br>(566 to 1,081)
70%	80%	0%	70%	77.3%<br>(61.8% to 82.7%)	1.3<br>(-1.3 to 4.2)	5.86<br>(4.86 to 8.50)	-0.15<br>(-0.26 to -0.12)	754	778<br>(620 to 1,126)
30%	80%	0%	30%	71.1%<br>(56.8% to 77.8%)	7.5<br>(3.9 to 10.0)	5.46<br>(4.69 to 7.91)	-0.56<br>(-0.88 to -0.46)	559	977<br>(811 to 1,415)
C. According to coverage at 14 yrs old irrespective of vaccination status and lower 1-dose efficacy (VE_1=85%, VD_1=Lifelong, VC_1=80%)									
Coverage at 14 yrs (irrespective of vaccination status):									
70%	94%	38%	56%	86.1%<br>(68.6% to 91.6%)	-7.5<br>(-9.0 to -3.8)	6.49<br>(5.48 to 9.40)	0.48<br>(0.41 to 0.74)	754	862<br>(717 to 1,246)
30%	86%	62%	24%	75.2%<br>(59.3% to 80.6%)	3.4<br>(0.5 to 5.9)	5.74<br>(4.74 to 8.25)	-0.27<br>(-0.48 to -0.23)	559	1,027<br>(783 to 1,476)

Vaccination scenarios	Proportion of girls vaccinated with			RR of cervical cancer incidence at equilibrium	Percentage point difference vs current 2-dose	Cases averted (millions)	Difference in cases averted vs current 2-dose (millions)	Total number of doses (millions)	Cases averted/ 100 000 doses
	≥ 1 dose	1 dose only	2 doses	Mean (80% UI[+])	Mean (80% UI)	Mean (80% UI)	Mean (80% UI)		Mean (80% UI)
**Sensitivity analysis for extended schedules with shorter duration of 1-dose protection** (VE_1=100%, VD_1=20 yrs, VC_1=80%)									
Coverage at 14yrs among previously vaccinated girls only:									
80%	80%	0%	80%	78.6% (62.5% to 84.3%)	0.0 (0.0 to 0.0)	6.02 (4.96 to 8.75)	0 (0 to 0)	802	750 (572 to 1,091)
70%	80%	0%	70%	76.5% (58.6% to 80.7%)	2.1 (0.5 to 5.8)	5.79 (4.81 to 8.38)	-0.22 (-0.40 to -0.16)	754	769 (597 to 1,111)
30%	80%	0%	30%	63.3% (50.0% to 67.4%)	15.3 (10.8 to 19.6)	4.83 (4.05 to 6.86)	-1.18 (-1.99 to -0.92)	559	865 (686 to 1,227)
Coverage at 14yrs among irrespective of vaccination status:									
70%	94%	38%	36%	81.4% (64.5% to 87.0%)	-2.8 (-4.8 to 2.0)	6.23 (5.35 to 8.91)	0.22 (0.08 to 0.30)	754	827 (669 to 1,182)
30%	86%	62%	24%	65.6% (50.4% to 71.1%)	13.0 (9.4 to 18.1)	5.03 (4.24 to 7.11)	-0.98 (-1.79 to -0.77)	559	900 (711 to 1,272)
**Sensitivity analysis for extended schedules with lower coverage at 9 years old (VC_1=65%)**									
Current 2-dose schedule (2 doses at 9 years old, VC=65%)	65%	0%	65%	64.5% (52.0% to 71.3%)	14.1 (8.9 to 17.5)	4.97 (4.11 to 7.16)	-1.05 (-1.57 to -0.87)	671	741 (610 to 1,068)
Coverage at 14yrs among irrespective of vaccination status:									
65% (VE_1=85%)	88%	46%	42%	79.5% (61.5% to 84.5%)	-0.9 (-3.6 to 3.7)	6.00 (5.02 to 8.63)	-0.02 (-0.15 to 0.05)	652	920 (738 to 1,323)
50% (VE_1=85%)	83%	50%	33%	73.8% (58.6% to 79.4%)	4.8 (1.8 to 8.7)	5.59 (4.62 to 8.03)	-0.43 (-0.69 to -0.35)	579	966 (739 to 1,387)
65% (VD_1=20 yrs)	88%	46%	42%	78.7% (62.5% to 84.1%)	-0.1 (-1.4 to 4.2)	5.98 (5.03 to 8.58)	-0.03 (-0.21 to -0.03)	652	918 (698 to 1,315)
50% (VD_1=20 yrs)	83%	50%	33%	70.2% (54.5% to 75.0%)	8.3 (5.6 to 12.6)	5.38 (4.48 to 7.65)	-0.64 (-1.13 to -0.53)	579	929 (706 to 1,321)

VE_i=vaccine efficacy of dose i. VD_i=vaccine duration of protection of dose i.VC_i=vaccination coverage of dose i.

[+]UI : uncertainty interval (80% UI: 10th-90th percentiles of the 50 parameter sets) and mean of the 10 best fitting parameter sets to the incidence of cervical cancer from Global Cancer Observatory 2020. Of note, uncertainty intervals should not be interpreted as confidence interval from a statistical point of view. Uncertainty intervals reflect uncertainty in model parameters and variability in sexual behaviour and HPV epidemiology within a country. To compare the results between vaccination strategies the uncertainty intervals around the following outcomes should be used: Percentage point difference in RR of cervical cancer vs current 2-dose and Difference in cases averted vs current 2-dose.

*Maximum/minimum value is the mean of the 10 best fitting parameter sets to the incidence of cervical cancer from Global Cancer Observatory 2020.

Figure S1. Projected population-level impact of five-year extended two-dose schedule, with one-dose duration of protection of 20 years and second dose given to previously vaccinated girls only.

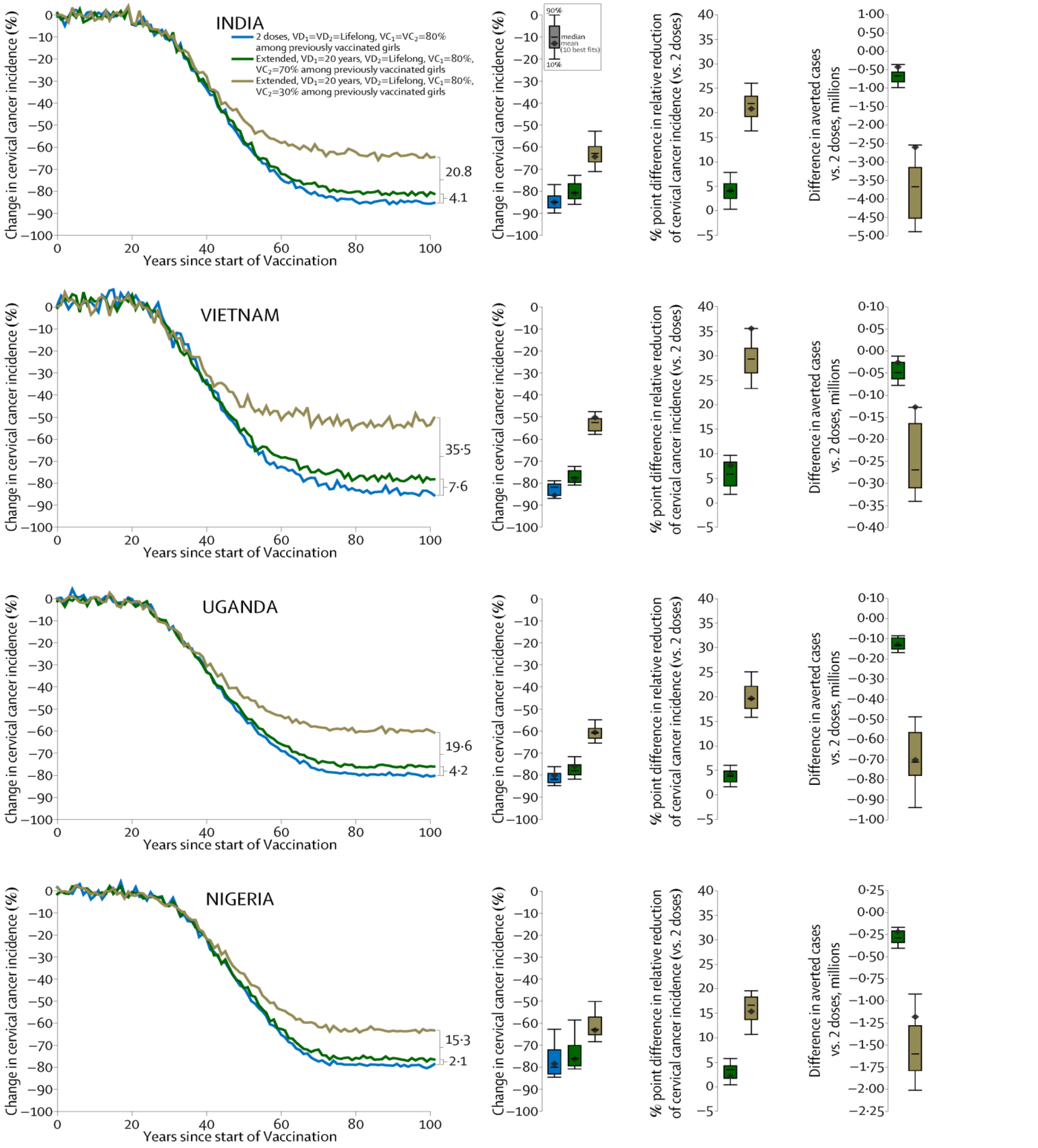

In extended scenarios, the dose at 14 years old is given to previously vaccinated girls only. VD_i=vaccine duration of protection of dose i. VC_i=vaccination coverage of dose i. Vaccine efficacy after one dose=100% for all scenarios. The line represents the mean of the 10 best fitting parameter sets to the incidence of cervical cancer from Global Cancer Observatory 2020. Boxplots: lower and upper limits: 10th and 90th percentiles of the 50 parameter sets, box: 25th and 75th percentiles of the 50 parameter sets, line: median of the 50 parameter sets, diamond: mean of the 10 best fitting parameter sets to the incidence of cervical cancer from Global Cancer Observatory 2020. Of note, uncertainty intervals should not be interpreted as confidence interval from a statistical point of view. Uncertainty intervals reflect uncertainty in model parameters and variability in HPV epidemiology within a country. To compare the results between vaccination strategies, the uncertainty intervals around the following outcomes should be used: Percentage point difference in relative reduction of cervical cancer (vs 2-dose) and Difference in averted cases (vs 2-dose).

Figure S2. Projected population-level impact of five-year extended two-dose schedule, with one-dose duration of protection of 20 years and second dose given at 14 years old, irrespective of vaccination status.

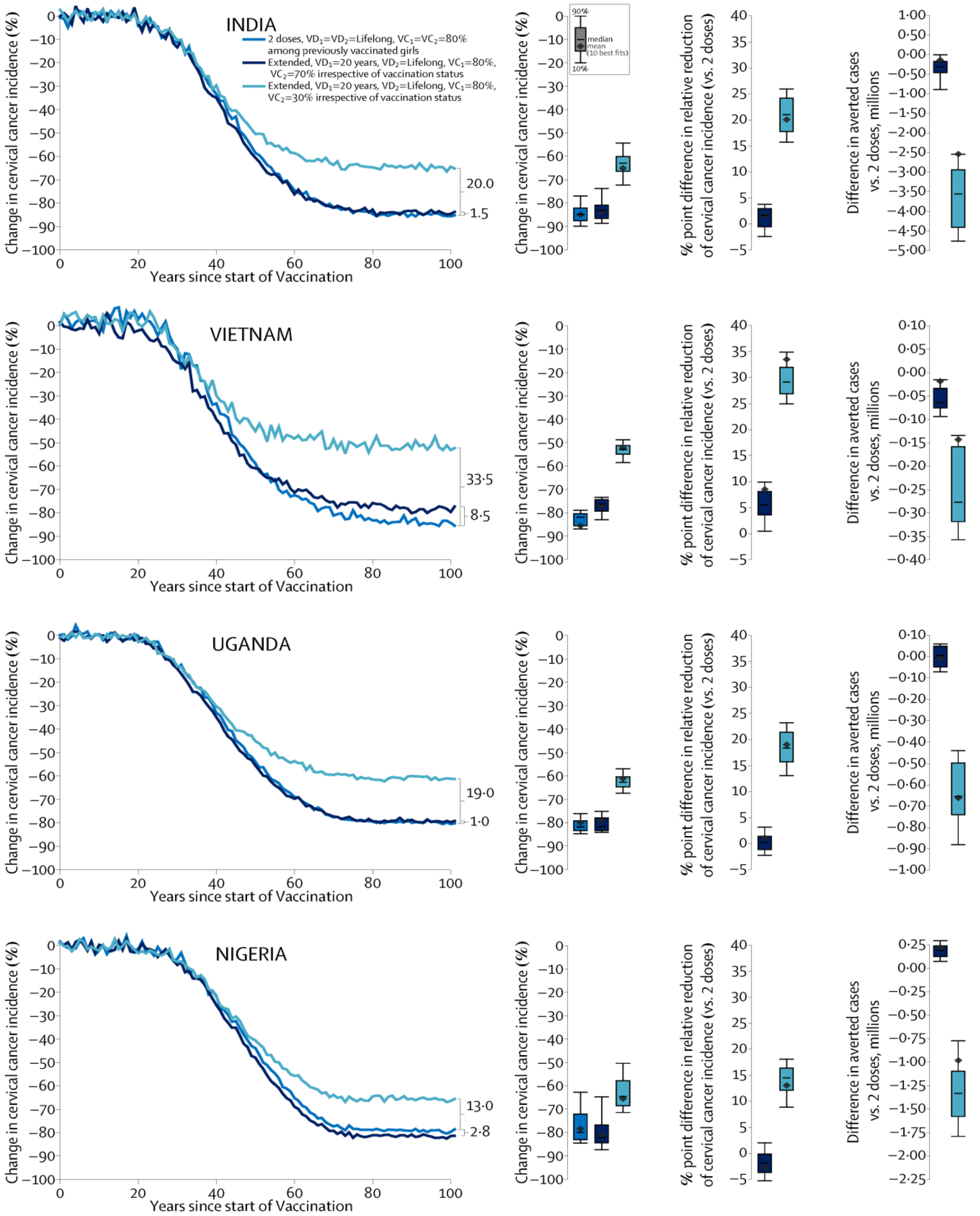

In extended scenarios, the dose at 14 years old is given to girls irrespective of vaccination status. VD_i=vaccine duration of protection of dose i. VC_i=vaccination coverage of dose i. Vaccine efficacy after one dose=100% for all scenarios. The line represents the mean of the 10 best fitting parameter sets to the incidence of cervical cancer from Global Cancer Observatory 2020. Boxplots: lower and upper limits: 10th and 90th percentiles of the 50 parameter sets, box: 25th and 75th percentiles of the 50 parameter sets, line: median of the 50 parameter sets, diamond: mean of the 10 best fitting parameter sets to the incidence of cervical cancer from Global Cancer Observatory 2020. Of note, uncertainty intervals should not be interpreted as confidence interval from a statistical point of view. Uncertainty intervals reflect uncertainty in model parameters and variability in HPV epidemiology within a country. To compare the results between vaccination strategies, the uncertainty intervals around the following outcomes should be used: Percentage point difference in relative reduction of cervical cancer (vs 2-dose) and Difference in averted cases (vs 2-dose).

Figure S3. Projected efficiency of the current two-dose schedule and five-year extended two-dose schedule with duration of one-dose protection of 20 years.

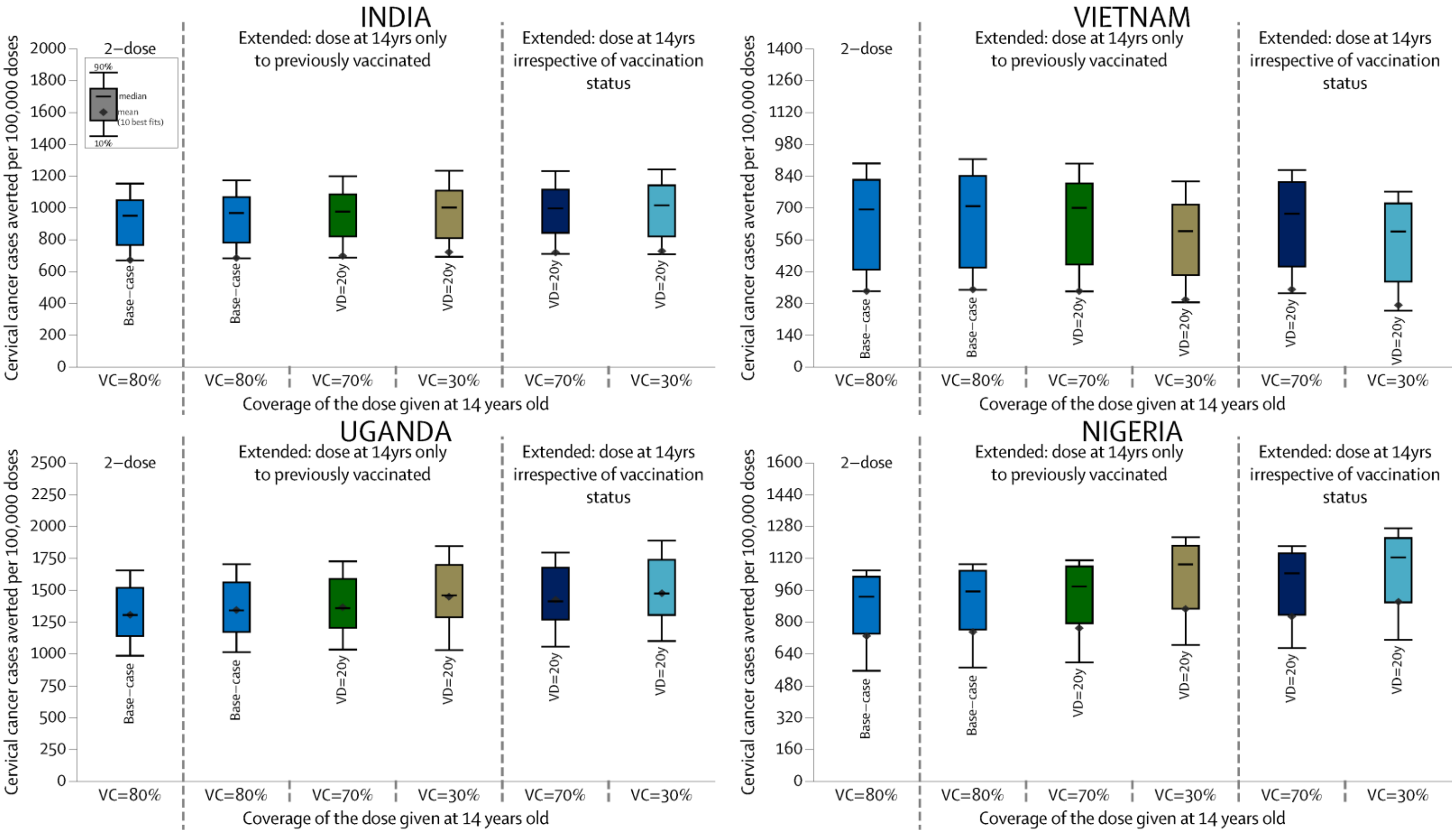

Base-case: VE=100%, VD=Lifelong.

VE=vaccine efficacy. VD=vaccine duration of protection. VC=vaccination coverage.

Projections are the mean of the 10 best fitting parameter sets to the incidence of cervical cancer from Global Cancer Observatory 2020. Boxplots: lower and upper limits: 10th and 90th percentiles of the 50 parameter sets, box: 25th and 75th percentiles of the 50 parameter sets, line: median of the 50 parameter sets, diamond: mean of the 10 best fitting parameter sets to the incidence of cervical cancer from Global Cancer Observatory 2020. Of note, uncertainty intervals should not be interpreted as confidence interval from a statistical point of view. Uncertainty intervals reflect uncertainty in model parameters and variability in HPV epidemiology within a country.

Figure S4. Projected population-level impact of five-year extended two-dose schedule, assuming 65% vaccination coverage at 9 years old, one-dose vaccine efficacy of 85% and second dose given at 14 years old, irrespective of vaccination status.

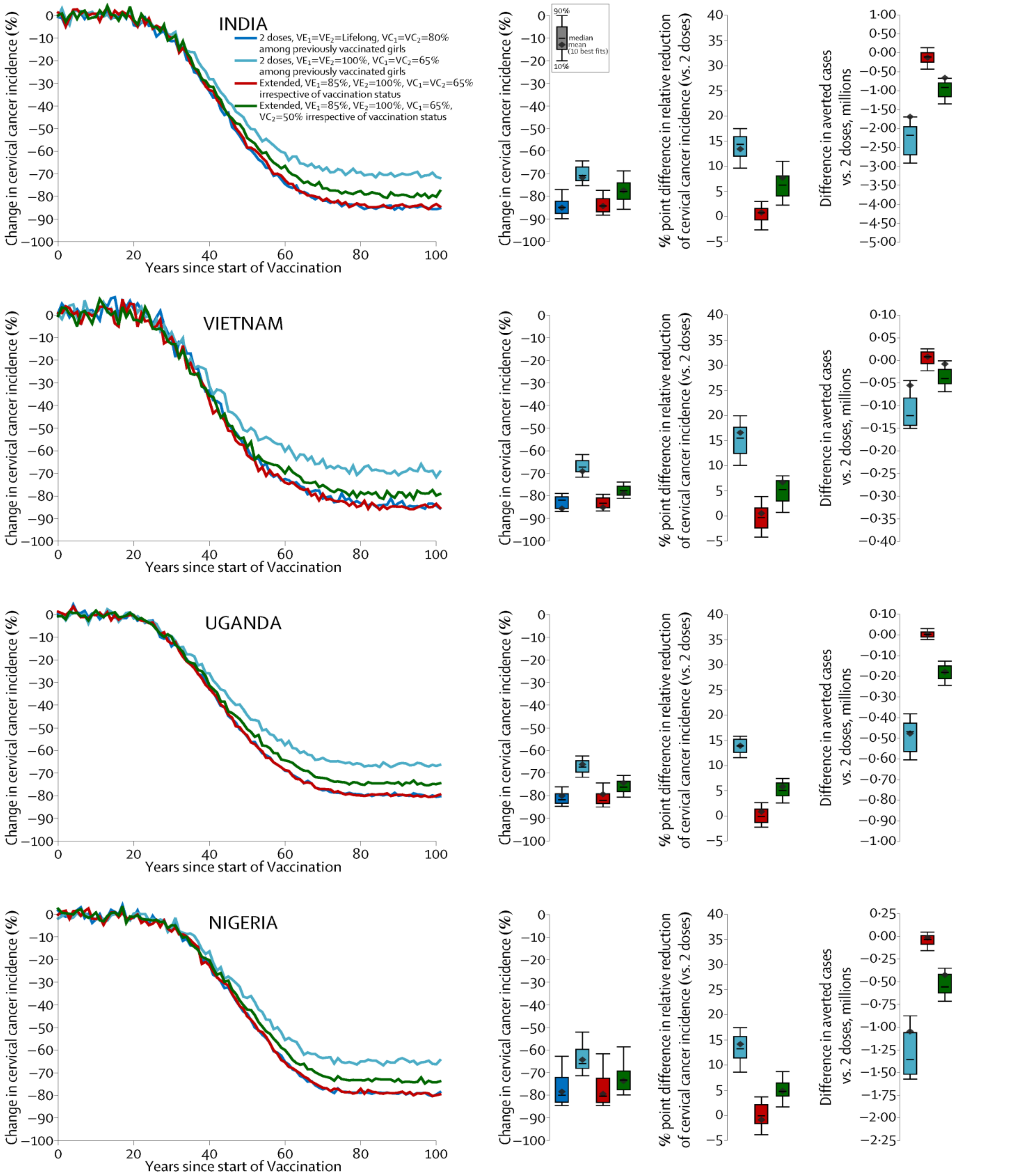

In extended scenarios, the dose at 14 years old is given to girls irrespective of vaccination status. VEi=vaccine efficacy of dose i. VCi=vaccination coverage of dose i. Vaccine duration of protection after one dose=Lifelong for all scenarios. The line represents the mean of the 10 best fitting parameter sets to the incidence of cervical cancer from Global Cancer Observatory 2020. Boxplots: lower and upper limits: 10th and 90th percentiles of the 50 parameter sets, box: 25th and 75th percentiles of the 50 parameter sets, line: median of the 50 parameter sets, diamond: mean of the 10 best fitting parameter sets to the incidence of cervical cancer from Global Cancer Observatory 2020. Of note, uncertainty intervals should not be interpreted as confidence interval from a statistical point of view. Uncertainty intervals reflect uncertainty in model parameters and variability in HPV epidemiology within a country. To compare the results between vaccination strategies, the uncertainty intervals around the following outcomes should be used: Percentage point difference in relative reduction of cervical cancer (vs 2-dose) and Difference in averted cases (vs 2-dose).

Figure S5. Projected population-level impact of five-year extended two-dose schedule, assuming 65% vaccination coverage at 9 years old, one-dose duration of protection of 20 years and second dose given at 14 years old, irrespective of vaccination status.

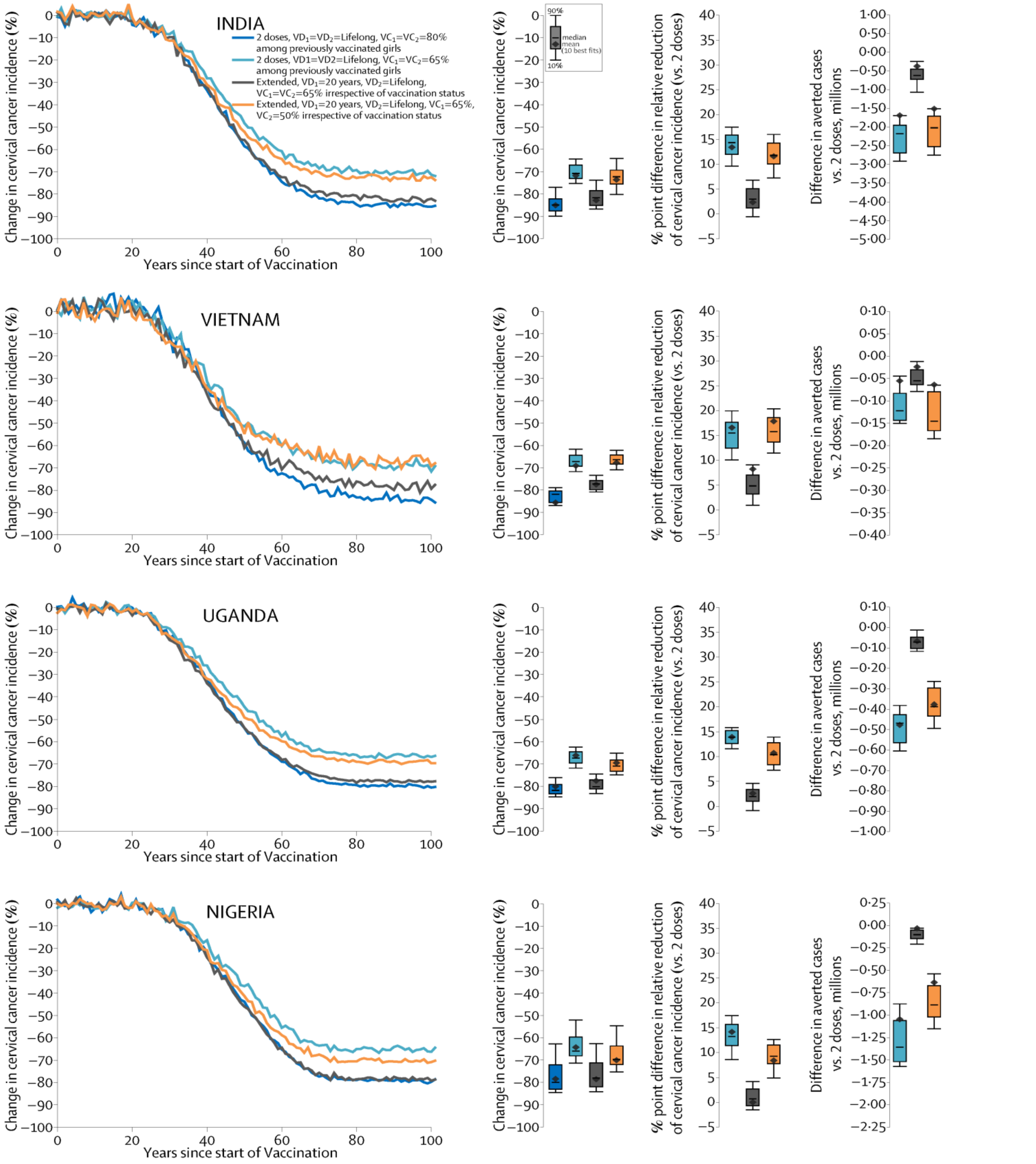

In extended scenarios, the dose at 14 years old is given to girls irrespective of vaccination status. VD_i=vaccine duration of protection of dose i. VC_i=vaccination coverage of dose i. Vaccine efficacy after one dose=100% for all scenarios. The line represents the mean of the 10 best fitting parameter sets to the incidence of cervical cancer from Global Cancer Observatory 2020. Boxplots: lower and upper limits: 10th and 90th percentiles of the 50 parameter sets, box: 25th and 75th percentiles of the 50 parameter sets, line: median of the 50 parameter sets, diamond: mean of the 10 best fitting parameter sets to the incidence of cervical cancer from Global Cancer Observatory 2020. Of note, uncertainty intervals should not be interpreted as confidence interval from a statistical point of view. Uncertainty intervals reflect uncertainty in model parameters and variability in HPV epidemiology within a country. To compare the results between vaccination strategies, the uncertainty intervals around the following outcomes should be used: Percentage point difference in relative reduction of cervical cancer (vs 2-dose) and Difference in averted cases (vs 2-dose).

Figure S6. Projected efficiency of the current two-dose schedule and five-year extended two-dose schedule, assuming 65% vaccination coverage at 9 years old.

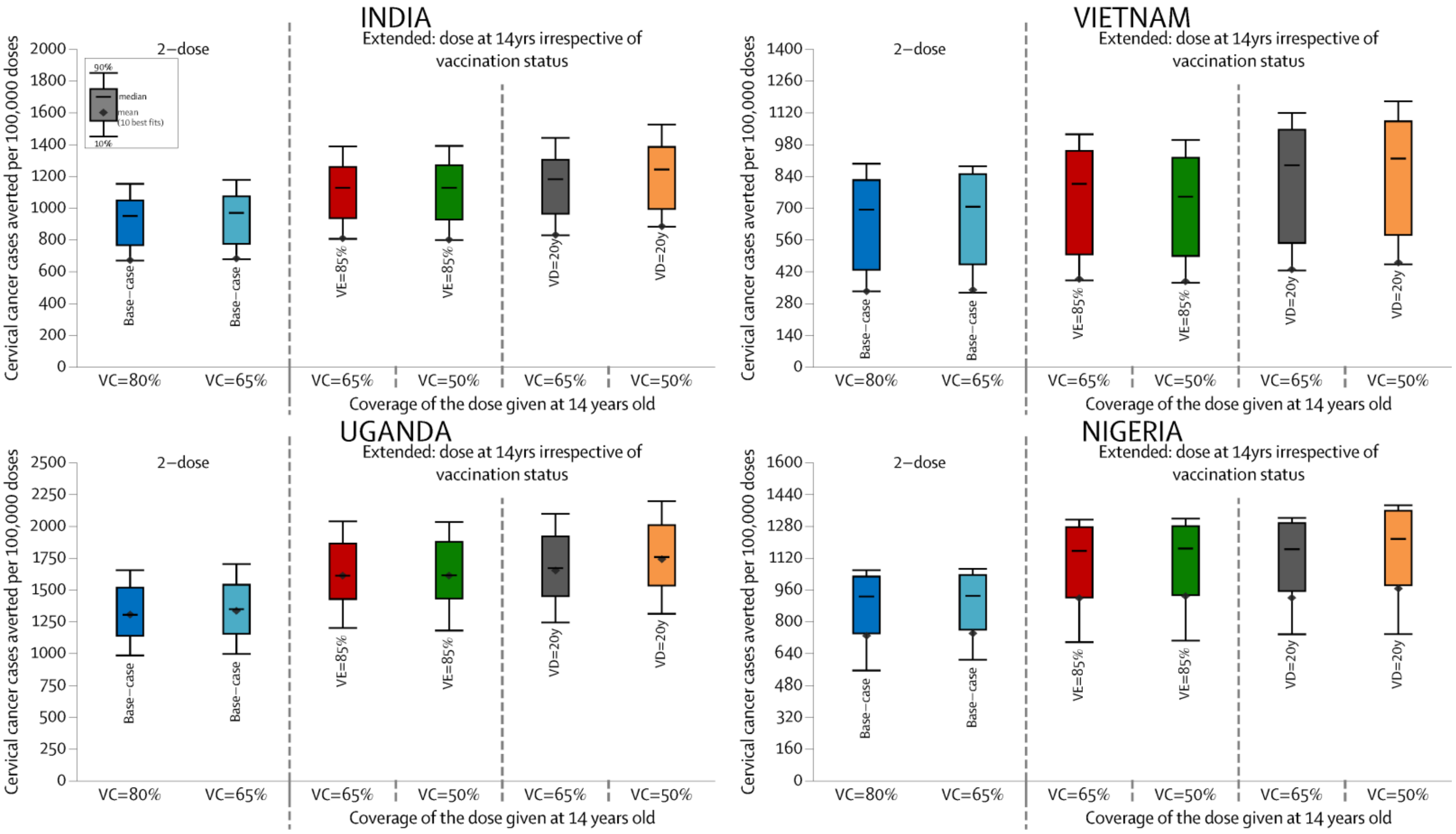

Base case: vaccine efficacy=100%, duration of protection=Lifelong.

VE=vaccine efficacy. VD=vaccine duration of protection. VC=vaccination coverage.

Projections are the mean of the 10 best fitting parameter sets to the incidence of cervical cancer from Global Cancer Observatory 2020.

Boxplots: lower and upper limits: 10th and 90th percentiles of the 50 parameter sets, box: 25th and 75th percentiles of the 50 parameter sets, line: median of the 50 parameter sets, diamond: mean of the 10 best fitting parameter sets to the incidence of cervical cancer from Global Cancer Observatory 2020. Of note, uncertainty intervals should not be interpreted as confidence interval from a statistical point of view. Uncertainty intervals reflect uncertainty in model parameters and variability in HPV epidemiology within a country.

Estimation of country-specific population size between 2100-2120

The age-stratified population for all countries between 2020 and 2100 were taken from United Nations World Population Prospects: The 2017 Revision (using the medium variant projections; medium-fertility assumption, normal mortality and normal international migration). Because the model projections of cervical cancer cases averted were until 2120 and population data were only available up to 2100, we extrapolated the United Nations World Population from 2100 to 2120.

To do this, first, we defined a population matrix (Pa,y) representing the number of people of age group “a” (five-year age groups) at year “y” (between 2000-2100). Second, we defined the effective survival rates ((Sa,y)= (Pa+1,y)/ (Pa,y-5)) as the ratio of the population of the subsequent age group over the population of the age group five years before. The effective birth rate ((B0-4,y)=(P0-4,y)) was defined as the 0-4 years old population. As survival and birth rates oscillate over time with different periods, we used Fourier analysis in the extrapolation process. The extrapolation of survival and birth rates after 2100 were performed in three steps: 1) for each age group, we removed the secular trend using a least-squares linear fit; 2) we performed a fast Fourier transform (FFT) and find local maxima in the power spectrum (dominant oscillatory components that have particular frequencies) that allowed us to define a least-squares fit (which is the sum of cosine functions representing each particular dominant frequency); and 3) we re-added the secular trend that was previously removed to these oscillatory components to get the full extrapolation results. Using this method, we estimated the effective survival rates and the birth rate for years 2100 onwards for all age groups and countries. To get the projections for the population for years 2101 to 2120, we used the birth rates and the effective survival rates ((P5-9,y)=(B0-4,y-5)·(S0-4,y)). Then, subsequent age group populations were obtained iteratively as ((Pa+1,y)=(Pa,y-5)·(Sa,y)).
